# Signaling networks and MiRNA crosstalk in ovarian cancer chemoresistance

**DOI:** 10.1186/s13048-025-01770-8

**Published:** 2025-08-14

**Authors:** Raksha Nayak, Samyak Pandey, Dileep Kumar, Sachindra Kumar, K Sreedhara Ranganath Pai

**Affiliations:** 1 Department of Pharmacology, Manipal College of Pharmaceutical Sciences, Manipal Academy of Higher Education, Manipal, 576104 India; 2 Department of Pharmaceutical Chemistry, Manipal College of Pharmaceutical Sciences, Manipal Academy of Higher Education, Manipal, 576104 Karnataka India

**Keywords:** Chemotherapy, Drug resistance, MiRNA, Ovarian cancer, Recurrence, Signaling pathway, Tumorigenesis

## Abstract

Epithelial ovarian cancer (EOC), accounting for 90–95% of all ovarian cancer (OC) cases, is the most lethal gynaecological malignancy, primarily due to late-stage diagnosis and the development of chemoresistance. While initial responses to Platinum- and Taxane-based chemotherapy are favorable, nearly 70% of patients relapse within five years. Although signaling pathways such as PI3K/AKT, MAPK, NF-κB, Notch, and Wnt/β-catenin have been individually studied in the context of chemoresistance, recent evidence highlights the importance of dynamic feedback loops and crosstalk among these networks in sustaining the resistant phenotype. Moreover, dysregulated microRNAs (miRNAs), as post-transcriptional regulators, fine-tune these pathways, creating self-sustaining circuits that promote drug efflux, inhibit apoptosis, and maintain cancer stemness. Reciprocal regulation between miRNAs and signaling components establishes robust networks that amplify chemoresistant phenotypes. The review provides a comprehensive overview of the molecular mechanisms driving chemoresistance, emphasising critical elements of signalling pathways and associated miRNAs that contribute to resistance and may function as biomarkers or therapeutic targets to mitigate chemoresistance. To improve clinical outcomes, future research should focus on identifying resistance-associated miRNA signatures and targeting nodal points within miRNA-signaling networks, thereby enabling the development of personalized therapies to overcome drug resistance in EOC.

## Introduction

OC is among the most lethal gynaecological malignancies, accounting for significant cancer-associated morbidity and mortality among women worldwide. Histologically, OC encompasses a diverse group of tumors, among which EOC accounts for 90–95% of cases and exhibits a high recurrence rate. The remaining 5–10% of OC cases are non-epithelial subtypes which primarily include germ cell tumors and sex cord-stromal tumors and are associated with significantly lower recurrence rates after platinum-based chemotherapy [1]. High-grade serous ovarian carcinoma (HGSOC), the predominant EOC subtype, constitutes 70–80% of all EOC cases and is characterized by genomic instability, TP53 mutations, and frequent recurrence. Other subtypes include endometrioid, clear cell, mucinous, and low-grade serous carcinomas, each presenting distinct molecular features and therapeutic responses [2]. Despite advances in cytoreductive surgery and platinum-taxane combination chemotherapy, the long-term survival of patients remains limited due to high recurrence rates and the eventual emergence of chemoresistance. Approximately 80% of patients with advanced-stage EOC relapse within five years of diagnosis, highlighting an urgent need to better understand the underlying molecular determinants of resistance [3, 4].

Chemotherapy resistance can be characterized as either intrinsic or acquired. Intrinsic resistance arises from inherent gene expression profiles in chemo-naïve tumor cells, while acquired resistance develops due to various changes triggered by exposure to chemotherapeutic treatments [5]. There are four major types of recurrent EOC: Platinum-sensitive, Platinum-resistant, persistent, and refractory, the latter three having varying degrees of chemotherapy resistance [6]. Chemoresistance is driven by multiple adaptive mechanisms, including enhanced DNA damage repair (DDR), drug efflux, apoptotic evasion, epithelial–mesenchymal transition (EMT), and cancer stem cell (CSC) persistence [7, 8]. Central to these phenotypes are alterations in key signaling cascades such as PI3K/AKT/mTOR, MAPK, NF-κB, JAK/STAT, TGF-β, Wnt/β-catenin, Notch, and GAS6/AXL. These pathways not only orchestrate survival and proliferative signaling but also interact with the tumor microenvironment (TME) to aid immune evasion and stromal remodeling, further reinforcing resistance [9].

Emerging evidence also highlights the role of miRNAs, small non-coding RNAs that post-transcriptionally regulate gene expression, in mediating chemoresistance. Dysregulated miRNAs act as oncogenes or tumor suppressors and modulate key components of signaling networks that control drug efflux, apoptosis, EMT, and CSC traits [10, 11]. Notably, reciprocal feedback exists between miRNAs and these signaling pathways, creating self-sustaining regulatory circuits that potentiate resistance under chemotherapeutic stress.

Despite the availability of reviews focusing on signaling pathways and miRNAs in EOC, the integrated role of miRNA-signaling pathway crosstalk in shaping chemoresistance networks in EOC remains underexplored. This review aims to address this critical gap by synthesizing current literature on how reciprocal regulatory interactions between dysregulated signaling cascades and miRNAs construct robust feedback loops that underpin the chemoresistant phenotype. We specifically focus on how miRNAs act as both effectors and regulators of key signaling nodes involved in drug resistance, highlighting their potential as therapeutic targets or biomarkers. Elucidating these interconnected networks offers a systems-level understanding of EOC chemoresistance and identifies targetable nodes within miRNA–signaling circuitry that may be utilized to overcome therapeutic resistance [12–14].

## Mechanisms of EOC chemoresistance

**Fig. 1:**
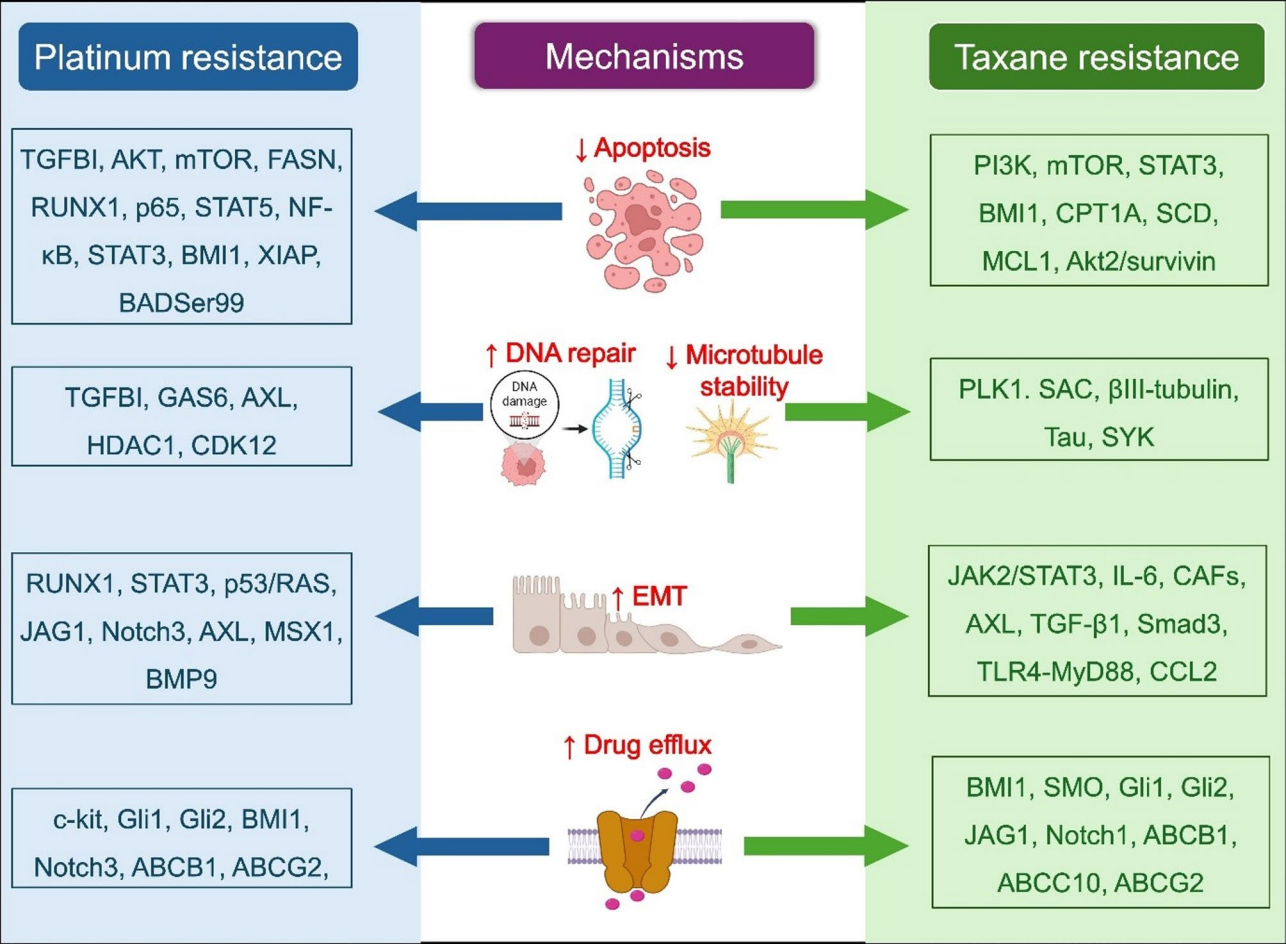
Molecular mechanisms and signaling components contributing to Platinum and Taxane resistance in EOC. The diagram illustrates key mediators associated with resistance to Platinum (left) and Taxane (right) chemotherapy agents. Central mechanisms include reduced apoptosis, enhanced DNA repair, decreased microtubule stability, EMT, and increased drug efflux. TGFBI, Transforming Growth Factor Beta-Induced protein; FASN, Fatty Acid Synthase; RUNX1, Runt-Related Transcription Factor 1; BMI1, B Lymphoma Mo-MLV Insertion Region 1 Homolog; HDAC1, Histone Deacetylase 1; XIAP, X-linked Inhibitor of Apoptosis Protein; CDK12, Cyclin-Dependent Kinase 12; MSX1, Msh Homeobox 1; BMP9, Bone Morphogenetic Protein 9; JAG1, Jagged Canonical Notch Ligand 1; CPT1A, Carnitine Palmitoyltransferase 1 A; SCD, Stearoyl-CoA Desaturase; MCL1, Myeloid Cell Leukemia 1; PLK1, Polo-Like Kinase 1; SYK, Spleen Tyrosine Kinase; CCL2, C-C Motif Chemokine Ligand 2; CAFs, Cancer-Associated Fibroblasts; TLR4, Toll-Like Receptor 4; SAC, Spindle Assembly Checkpoint; MyD88, Myeloid Differentiation Primary Response 88

Chemoresistance remains a principal challenge in the therapeutic management of EOC, particularly in patients undergoing Platinum and Taxane-based therapies. Tumor cells employ various adaptive mechanisms to evade the cytotoxic effects of drugs, contributing to high rates of relapse and poor prognosis. Notably, components associated with signaling pathways, such as PI3K/AKT/mTOR, MAPK, JAK/STAT, and NF-κB, play a critical role in triggering chemoresistance through these mechanisms (Fig. 1). The exploration of these pathways reveals their intricate involvement in the development of resistance to chemotherapy.

One of the key mechanisms for chemoresistance is the suppression of apoptosis. In EOC, apoptosis is often disrupted, allowing malignant cells to evade basal cell mechanisms and continue proliferating. The interplay between the activators and inhibitors of apoptosis can influence chemotherapy-induced cell death, leading to intrinsic or acquired chemotherapy resistance [15]. Inhibitors of Apoptosis (IAPs) have been reported to be dysregulated in EOC, with the overexpression of X-linked IAPs (XIAPs) being associated with resistance to Platinum-based therapies [16]. Dysregulation of signaling molecules like SCD, CPT1A, MCL1, and AKT2/survivin was observed in Paclitaxel-resistant EOC [17–19].

Platinum compounds form DNA cross-links and adducts between DNA and proteins, resulting in DNA damage and cell death. Impaired DDR activates senescence or apoptotic signals within cells, whereas aberrant DDR activation promotes cancer cell survival and resistance to chemotherapy and PARP inhibitors [20]. miRNAs and signaling molecules are known to interact with components of the DDR machinery to promote chemoresistance [21]. Upregulation of DDR-related regulators such as GAS6, AXL, HDAC1, and CDK12 restores genomic integrity following chemotherapy-induced damage, allowing cancer cell survival [22, 23].

Taxanes interact with the β-tubulin microtubule subunit, leading to dysregulation of its function and cell cycle arrest. Taxane resistance is closely linked to alterations in microtubule-associated proteins (MAPs), MAP kinases (MAPKs), and tubulin isotypes. These alterations impair Taxane binding, disrupt mitotic spindle assembly, and allow cells to evade drug-induced mitotic arrest [24]. βIII-tubulin, a key tubulin isotype, exhibits reduced binding affinity to Paclitaxel and is frequently overexpressed in Taxane-resistant cells [25]. Proteins like tau and SYK interfere with Taxane binding and microtubule dynamics, promoting resistance [26, 27]. It was found that Taxane resistance in EOC is reduced, and mitotic escape was prevented by the activation of the spindle assembly checkpoint (SAC) through PLK1 inhibition [28]. Such components can potentially serve as resistance mediators, biomarkers, or therapeutic targets in overcoming Taxane resistance.

EMT is a hallmark of cancer cells wherein epithelial cells acquire mesenchymal properties, enhancing their motility and invasiveness. This phenotypic switch involves reduced expression of epithelial markers, including E-cadherin, alongside increased expression of mesenchymal markers like N-cadherin, fibronectin, and vimentin, fostering a more aggressive and chemotherapy-resistant tumor phenotype [29]. The mesenchymal-like cancer cells exhibit enhanced activation of survival signaling cascades such as PI3K/AKT, NF-κB, and TGF-β/Smad, which attenuate drug-induced apoptosis. EMT is also linked to the development of cancer stem cell (CSC)-like characteristics, which confer intrinsic resistance to chemotherapeutic agents. Furthermore, several transcription factors that orchestrate EMT, such as Snail, Slug, Twist1, and ZEB1/2, are also implicated in the regulation of genes responsible for DDR, cell cycle arrest, and drug metabolism [30]. Dysregulation of MSX1, Smad3, TLR-4-MyD88, and CCL2 has been reported to promote Platinum and Taxane resistance in EOC by inducing EMT [31–34].

Increased drug efflux is a major mechanism of chemoresistance in EOC, primarily mediated by members of the ATP-binding cassette (ABC) transporter family, including P-glycoprotein (ABCB1), ABCC1, and ABCG2. These transporters reduce intracellular drug levels to below cytotoxic thresholds by actively pumping out chemotherapeutic agents, thereby promoting chemoresistance. The activation of Notch, Wnt, and Hedgehog pathways and dysregulation of specific miRNAs in EOC contribute to the transcriptional and post-transcriptional upregulation of these efflux transporters [35–37].

## Major signaling pathways in EOC chemoresistance

**Fig. 2:**
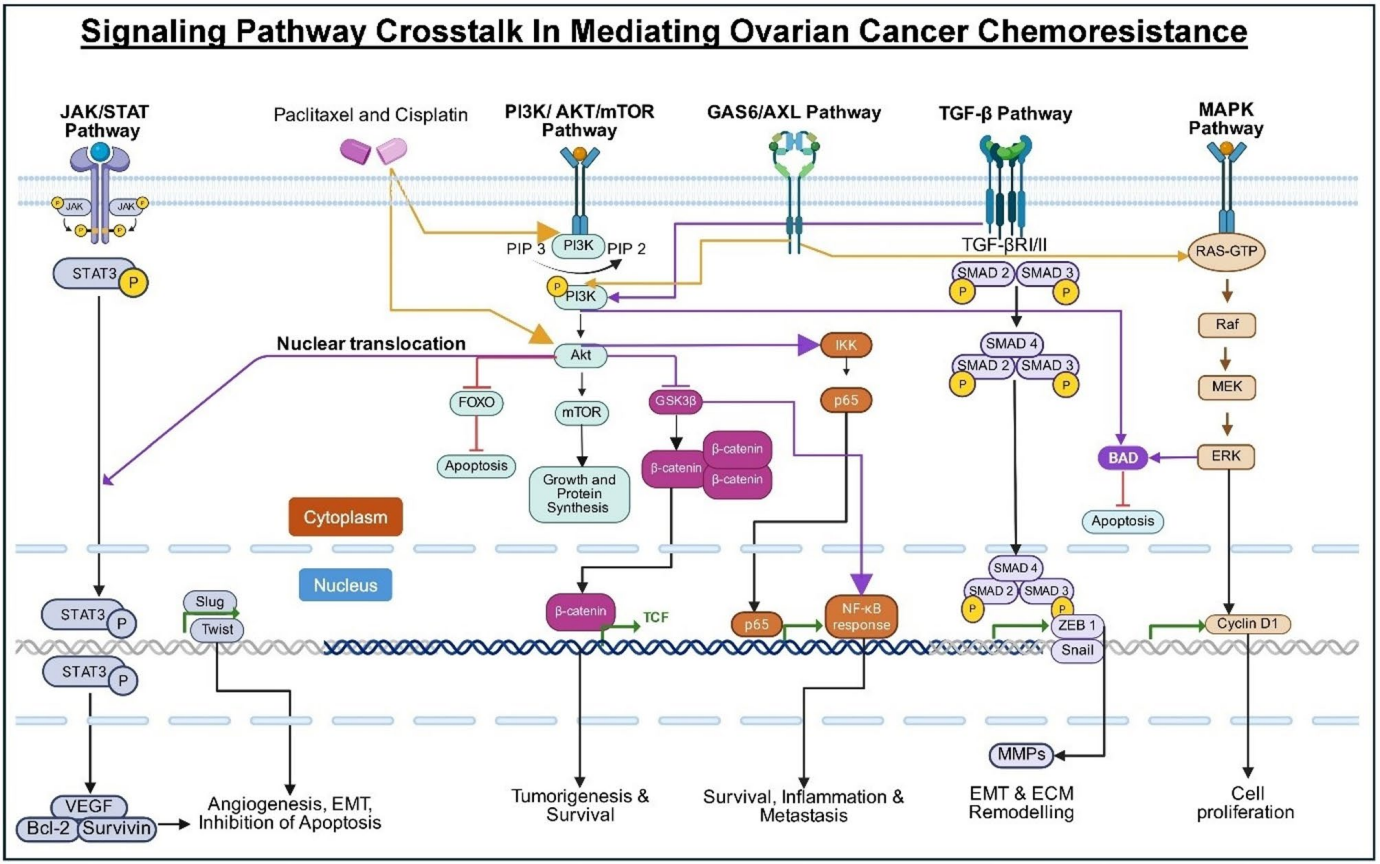
Crosstalk among major signaling pathways mediating chemoresistance in EOC. The diagram illustrates the complex interplay between major signaling pathways in promoting chemoresistance in EOC. Activation of these pathways drives tumor-promoting processes like EMT, apoptosis inhibition, angiogenesis, extracellular matrix remodeling, inflammation, and uncontrolled proliferation. Key components that serve as convergence points represent promising targets for therapeutic intervention, aiming to overcome resistance through the inhibition of multiple resistance-driving pathways

Several signaling pathways are deregulated in EOC, allowing tumor cells to evade apoptosis, adapt to stress, and develop resistance to chemotherapy. Understanding the molecular architecture of these signaling cascades is essential for identifying critical nodes that contribute to chemoresistance (Fig. 2). Signaling pathways implicated in EOC chemoresistance play significant roles in sustaining malignancy and present potential therapeutic targets (Table 1).

**Table 1:** Pathway-specific components and their role in EOC chemoresistance

Component	Mechanism	Subtype	Resistant drug	Modulators (Stage of investigation)	Reference
**PI3K/ AKT pathway**					
*TGFBI*	Reduces apoptosis and induces DDR pathways	Ovarian Serous Cystadenocarcinoma	Cisplatin	siRNAs (in vitro)	[45]
AKT	Phosphorylation of AKT at S473 by DNA-dependent protein kinase (DNA-PK)	HGSOC	Platinum compounds	AKT isoform specific siRNAs (in vitro)	[180]
AKT	Downregulation of apoptosis-initiating factor (AIF)	HGSOC	Platinum compounds	siRNAs (in vitro)	[181]
PI3K/mTOR	Increases cell proliferation and reduces apoptosis and G_1_ cell cycle arrest	Ovarian endometroid adenocarcinoma	Paclitaxel and Platinum compounds	PI3K/mTOR dual inhibitor CMG002 (in vitro and in vivo)	[182]
FASN	Reduces apoptosis	HGSOC	Platinum compounds	Cerulenin and C75 (in vitro)	[52]
*FOXO1*	Modulation of oxidative stress by regulation of MnSOD	LGSOC	Paclitaxel	siRNAs (in vitro)	[183]
*UBE2S*	Inhibition of autophagy by activating PI3K/AKT/mTOR pathway	EOC	Cisplatin	shRNAs (in vitro and in vivo)	[184]
**NF-κB Pathway**					
RUNX1	Apoptosis suppression and EMT	HGSOC	Cisplatin	*RUNX1* inhibitor Ro5-3335 (in vitro)	[185]
RELA and STAT5	Upregulation of anti-apoptotic protein Bcl-xL	HGSOC	Carboplatin	shRNAs, BMS-345,541 (NF-κB inhibitor), and Dasatinib (STAT5 inhibitor) (in vitro)	[63]
NF-κB	Upregulation of cellular inhibitors of Apoptosis Protein-1 (cIAP1)	HGSOC	Cisplatin	Bay-117,082 (in vitro)	[186]
**JAK/STAT pathway**					
*FBN1*	Glycolysis and angiogenesis via FBN1/VEGFR2/STAT2 activation	HGSOC	Cisplatin	*FBN1*-knockout models (in vitro and in vivo)	[72]
STAT3	Reduces chemotherapy-induced apoptosis and increases Bcl-xL, Bcl-2, and survivin levels	Endometroid and Clear cell carcinoma	Paclitaxel and Cisplatin	siRNA, Cryptotanshinone (STAT3 inhibitor) (in vitro)	[79, 187]
STAT3 and p53/RAS	Slug/ PI3K/MAPK/ AKT-mediated EMT and autophagy	EOC	Cisplatin	STAT3 constructs (in vitro and in vivo)	[84]
*L1CAM*	Stemness maintained via FGFR1/SRC/STAT3 signaling	HGSOC	Paclitaxel	CE7 (L1CAM neutralizing antibody) and Napabucasin (STAT3 inhibitor) (in vitro and in vivo)	[80]
*Wip1*	Reduces VEGF/STAT3-dependent mechanism	Serous EOC	Increases Platinum sensitivity	Stattic (STAT3 inhibitor) (in vitro)	[81]
Cancer associated fibroblasts	Induction of EMT via IL-6/JAK2/STAT3 axis activation	HGSOC	Paclitaxel	IL-6 neutralizing antibody, AG490 (JAK2/STAT3 inhibitor), SB431542 (TGF-β inhibitor) (in vitro and retrospective analysis of patient samples)	[85]
**TGF-β pathway**					
*ZEB1*	Upregulates of *SOX2*,* OCT4*,* NANOG*,* CD44*, and *CD117*	HGSOC	Cisplatin	siRNAs (in vitro)	[108]
*TGF-β1*	BRCA1/Smad3 signaling	HGSOC	Cisplatin	TGF-β1 overexpression (vectors) and siRNAs (in vitro and in vivo)	[110]
*Endoglin*	Upregulation of *BARD1*,* H2AFX*,* NBN*,* NTHL1*, and *SIRT1.*	EOC	Platinum compounds	siRNAs (in vitro and in vivo)	[188]
*DACH2*	Upregulation of Chek1, Chek2, MCM3 and Ki67	EOC	Cisplatin	siRNAs (in vitro and ex vivo)	[189]
BMP9	Enhanced EMT induction	Serous adenocarcinoma	Platinum compounds	Exogenous recombinant BMP9 (in vitro)	[190]
**MAPK Pathway**					
PBK	ERK/mTOR-mediated induction of autophagy	HGSOC	Cisplatin	U0126 (MEK inhibitor) and OTS514 (PBK inhibitor) (in vitro and in vivo)	[191]
MEK 1/2	Activation of mutant p53	HGSOC	Cisplatin	U0126 and PD 98,056 (MEK inhibitor) (in vitro)	[125]
MKP-1	Stimulation of PARP-1 and PAR	HGSOC	Cisplatin	SB203580 (p38 MAPK inhibitor), U0126, and SP600125 (JNK pathway inhibitor) (in vitro)	[131, 192]
**Notch Pathway**					
*Notch1*	Suppression of apoptosis and induction of EMT by upregulating Snail, N-cadherin, Vimentin, and MMP-2, and downregulating E-cadherin	EOC	Cisplatin	Lentiviral-mediated overexpression and knockdown of *Notch1* (in vitro)	[89]
*CCL20*	Upregulation of *Notch1*,* Oct4*,* CCR6*, and *ABCG1*	HGSOC	Paclitaxel	siRNAs (in vitro and in vivo)	[193]
γ-secretase	Upregulation of Notch signaling	HGSOC	Cisplatin	DAPT (γ-secretase inhibitor) (in vitro)	[91]
*JAG1*	Crosstalk with Gli2, upregulation of *ABCB1*	HGSOC	Taxanes	Anti-Jagged1 siRNA (in vitro and in vivo)	[92]
*Notch3*	Enhanced EMT via overexpression of *SUSD2*	HGSOC	Cisplatin	shRNAs for *SUSD2* and *Notch3* (in vitro and in vivo)	[96]​
*Notch3*	Upregulation of *ABCB1*,* Nanog*,* Oct4*,* Klf4*,* Rex1*,* Rif1*,* Sall4*, and *NAC1*	Recurrent HGSOC	Carboplatin	*Notch3* shRNA (in vitro and retrospective analysis of patient samples)	[37]
*JAG1*	Crosstalk with JAK/STAT3 to enhance EMT	HGSOC	Platinum compounds	DAPT, *Jagged1* siRNA/ shRNA (in vitro an`d in vivo)	[194]
*Notch3*	Expansion and maintenance of CSCs	Serous adenocarcinomas	Cisplatin	*Notch3* siRNA (in vitro and in vivo)	[195]
Notch3	Inhibition of ERK phosphorylation and EMT induction	Serous adenocarcinoma	Carboplatin	U0126, NICD3 overexpression (in vitro)	[196]
*NR2F6*	Sustained activation of Notch3 signaling	EOC	Cisplatin	RO4929097 (γ-secretase inhibitor) and *NR2F6* siRNA (in vitro and in vivo)	[197]
**Wnt/β-catenin Pathway**					
β-catenin	Increases CSC tumor sphere formation	HGSOC	Platinum compounds	iCG-001 (β-catenin/ CBP inhibitor) (in vitro, in vivo and ex vivo)	[198]
R-spondin 1	Activation of Wnt/β-catenin signaling	HGSOC	Cisplatin and Paclitaxel	Recombinant R-spondin 1 and Wnt3A proteins, *RSPO1* overexpression and siRNA knockdown (in vitro and ex vivo)	[139]
FoxM1	Positive feedback loop with β-catenin	HGSOC	Paclitaxel	Thiostrepton (FoxM1 inhibitor), FH535 (β-catenin inhibitor), *FoxM1* siRNA and plasmid overexpression (in vitro, in vivo and ex vivo)	[199]
c-kit	Activation of Wnt/β-catenin and ABCG2	HGSOC	Cisplatin and Paclitaxel	Imatinib, LY294002 (PI3K inhibitor), and PD98059 (MEK inhibitor) (in vitro and in vivo)	[35]
MMP − 10	Activation of canonical Wnt/β-catenin signaling by inhibition Wnt5A	Serous adenocarcinomas	Platinum compounds	MMP inhibitor NNGH (in vitro and in vivo)	[200]
**GAS6/AXL Pathway**					
GAS6	Enhanced DDR through homologous recombination	HR-proficient HGSOC	Platinum compounds	Batiraxcept (AVB-500) (in vitro, in vivo and ex vivo)	[147]
AXL	Increases glycolysis by phosphorylation of PKM2	HGSOC	Cisplatin	R428 (AXL inhibitor) (in vitro, in vivo and ex vivo)	[157]
AXL	Enhanced EMT and DDR mechanisms	HGSOC	Platinum and Taxane compounds	AXL inhibitor (BGB324/bemcentinib) and *AXL* shRNA knockdown (in vitro, in vivo and ex vivo)	[154]
GAS6/AXL	Increased expression of BRCA1/2 and RAD51	HGSOC	Carboplatin	Batiraxcept (AVB-500) (in vitro, in vivo and ex vivo)	[156]
**Hedgehog Pathway**					
Gli1	Upregulation of c-jun Ser63/73, ERCC1, XPD, and XRCC1	HGSOC	Cisplatin	Cyclopamine, anti-Gli shRNA (in vitro)	[163]
Gli2	Increased expression of ABCB1	HGSOC	Cisplatin	Cyclopamine, Verapamil, GANT61, and Gli2A (in vitro)	​​ [167, 201]
SMO, Gli1, and Gli2	Increased expression of ABCB1	HGSOC	Paclitaxel	Sonidegib, siRNAs for Gli1, Gli2, and SMO (in vitro and in vivo)	[165]
BMI1	Apoptosis suppression and upregulation of *SOX2*,* OCT4*,* KLF4*, and *ABCB1*	HGSOC	Carboplatin and Paclitaxel	PTC596 (Unesbulin; BMI1 inhibitor) (in vivo, currently in Phase 1 clinical trial (NCT03206645))	[202]

### PI3K/AKT/mTOR pathway

The PI3K/AKT/mTOR signaling pathway plays a pivotal role in the regulation of critical cellular processes and is a central contributor to the development of chemoresistance in cancer. This pathway is susceptible to dysregulation, mutations, and aberrant expression of associated signaling components, all of which enhance the resistance of cancer cells to therapy and eventually increase the progression of the disease.

The PI3K/AKT/mTOR pathway, which facilitates cell proliferation and migration, is often deregulated in EOC. Numerous preclinical studies have shown that chemoresistance, especially to Taxanes, stems from activation of PI3K and hyperactivating mutations [38]​. Abnormal activation of this pathway is known to enhance EMT and upregulate CSC markers, leading to chemoresistance [39]​. Mutations of phosphatidylinositol-4,5-bisphosphate 3-kinase catalytic subunit α (PIK3CA) and the subsequent overactivation of the pathway are more commonly observed in clear cell carcinomas than in serous or mucinous subtypes [40]​. The abnormal activation of the pathway inhibited chemotherapy-induced apoptosis through the upregulation of Bcl-2 and XIAP (anti-apoptotic genes), and the downregulation of Bax (pro-apoptotic gene), which contributed to multidrug resistance [41, 42]. The sustained activation of the PI3K/AKT-NF-κB axis upregulated P-glycoprotein expression and accelerated cyclin D-mediated cell cycle progression, leading to increased tumor aggressiveness and resistance to Cisplatin and Paclitaxel [43]​. The PI3K/AKT signaling pathway promoted multidrug resistance by inhibiting caspase-3 activity and suppressing apoptosis, while also contributing to miRNA dysregulation, further reinforcing chemoresistant phenotypes [44]​. The upregulation of the PI3K/AKT pathway by TGFBI increased Cisplatin resistance by reducing apoptosis and inducing DDR pathways [45]​. BADSer99 phosphorylation was identified as a key mediator of Cisplatin resistance in EOC, with its inhibition enhancing apoptosis and reducing IC₅₀ of Cisplatin in both sensitive and resistant cell lines. NPB, a potent and specific inhibitor of BAD at Ser99, demonstrated synergistic efficacy with Cisplatin and AKT inhibitor AZD5363, suppressing CSC-like properties and improving therapeutic outcomes in vitro and in vivo [46]​.

Increased AKT activation and mTOR phosphorylation contributed to primary Platinum resistance. Elevated AKT expression and activation enhanced resistance to Platinum-based chemotherapy and Paclitaxel, as evidenced by increased Cisplatin resistance in AKT-overexpressing cell lines and higher phospho-AKT levels in Paclitaxel-resistant cells [47]​. Activation of AKT is considered a critical modulator influencing Platinum resistance in immortalized, patient-acquired Platinum-resistant HGSOC [48]​. Patients with advanced chemoresistant EOC exhibited significant upregulation of p-p70S6K, a downstream target of mTOR [38]​. AKT inhibition restored Cisplatin sensitivity in Platinum-resistant EOC cells. AKT inhibitors, AZD8835 and AZD5363, enhanced the sensitivity of chemoresistant EOC cells to Paclitaxel and Cisplatin. However, only AZD5363 effectively suppressed *COL11A1* mRNA expression and promoter activity, key elements involved in AKT signaling and chemoresistance development [49]​. Dual inhibition of PI3K and mTOR by CMG002 reduced Paclitaxel and Cisplatin resistance by suppressing cell proliferation and inducing apoptosis and G1 cell cycle arrest [50]​.

Fatty acid synthase (FASN) enzyme is overexpressed in 80% of EOC cases and correlates with poor prognosis. Its tumorigenic role was attributed to its ability to stimulate PI3K/mTOR signaling [51]. It was observed that FASN inhibition reversed Cisplatin resistance by inducing apoptosis in Platinum-sensitive and resistant cell lines [52]​. The PI3K/AKT pathway negatively regulates FOXO1 expression, which is often downregulated in Platinum-resistant EOCs [53]​. Conversely, FOXO1 was implicated in mediating Paclitaxel-resistance through protein-protein interaction with Thioredoxin 1 [54]​.

GSK3β, a downstream component of the PI3K/AKT pathway, is a serine/threonine kinase often overexpressed in EOC, which plays a crucial role in mediating drug resistance through the PI3K/AKT pathway [55]​. AKT phosphorylates GSK3β, inactivating it, which leads to the cytoplasmic accumulation of β-catenin. The subsequent nuclear translocation of β-catenin promotes the expression of genes that augment cell proliferation (c-Myc, cyclin D1, and c-Jun) and multidrug resistance (MDR) (MRP1 and survivin) [56]​. Conversely, it was found that GSK3β regulates cell viability and induces MDR through the PTEN/PI3K/AKT pathway, where GSK3β induces PTEN phosphorylation, leading to AKT activation. The interplay between GSK3β and PTEN regulates the AKT activity through a feedback loop [57]​.

A Phase I clinical trial (CT) (NCT04586335) is assessing the safety and efficacy of a combination of CYH33, a PI3Kα inhibitor, and Olaparib for treating Platinum-resistant EOC, reflecting the clinical translation of targeting this pathway. The maximum tolerable dose of Capivasertib, a kinase inhibitor targeting all three isoforms of AKT, alone or combined with Bevacizumab and Paclitaxel, is under investigation in a Phase I CT (NCT05039801) for advanced solid tumors, including OC. A combination of Copanlisib (PI3K inhibitor) and Olaparib was evaluated in a Phase II CT (NCT05295589) compared to standard chemotherapy in recurrent Platinum-resistant OC following prior PARP inhibitor therapy. Another Phase II CT (NCT04374630) is assessing the efficacy of Paclitaxel and Afuresertib, an AKT inhibitor, in Platinum-resistant OC.

### NF-κB pathway

NF-κB exhibits a biphasic role, initially sensitizing cells to Platinum- and Taxane-induced apoptosis but later promoting tumor aggressiveness and chemoresistance [58]​. NF-κB-mediated chemoresistance can be classified into two types: primary, driven by intrinsic NF-κB activation within cancer cells, and acquired, triggered by chemotherapeutic agents such as Cisplatin, Carboplatin, Paclitaxel, and Doxorubicin, which activate downstream NF-κB signaling pathways [59]​.

Increased nuclear translocation of the p65 subunit and phosphorylation of IκB kinase, indicative of NF-κB activation, were observed in patients with chemoresistant EOC [60]​. Canonical NF-κB signaling promotes anti-apoptotic and immunomodulatory responses, while non-canonical NF-κB activation supports CSC maintenance and tumor re-initiation [61]​. Furthermore, the NF-κB signaling pathway contributes to immunosuppression and immune evasion cells partly through NF-κB-mediated production of IL-6. IL-6 impairs dendritic cells while promoting the production and homing of immune-inhibitory myeloid-derived suppressor cells (MDSCs) and IL-8, which in turn enhances the expression of the immunosuppressive enzyme arginase [62]​.

NF-κB activation enhanced tumor cell proliferation by stimulating the production of MMP9, IL-8, Bcl2, and TNF-α in the TME. NF-κB activation in patients with Platinum-resistant EOC was associated with poor prognosis [61] ​. The upregulation of the p65 RelA subunit of NF-κB and STAT5 increased Carboplatin chemoresistance [63]​. Administration of Dehydroxymethylepoxyquinomicin (DHMEQ), an NF-κB inhibitor, improved response to Platinum drugs by reversing immunosuppression and inducing apoptosis [64]​. The interaction of RelA with BRCA1 enhanced the translational activation of several NF-κB target genes. The activation of NF-κB by BRCA1 in response to DNA-damaging chemotherapeutic agents, including Etoposide and Camptothecin, induced chemoresistance by repressing BRCA1-mediated apoptosis in response to DNA damage [65]​. GSK3β also mediates chemoresistance through NF-κB pathway activation [66]​. Nuclear accumulation of GSK3β regulates histone modifications, facilitating NF-κB-mediated transcriptional activation of Bcl-2 and XIAP, which promotes cancer cell survival and chemoresistance [67].

It was observed that Cisplatin-resistant Caov-3 EOC cells exhibited higher basal levels of NF-κB activity and IκBα phosphorylation in comparison to Cisplatin-sensitive A2780 cells. Pharmacological suppression of NF-κB using BAY 11-7085, an IκBα phosphorylation inhibitor, enhanced Cisplatin-induced NF-κB inhibition, apoptosis, and reduced XIAP expression and invasive potential. These findings support NF-κB inhibition as a strategy to improve Cisplatin efficacy in resistant EOC [68]​. A similar study reported that Paclitaxel transiently activated NF-κB signaling through the PI3K/AKT/IKK/IκBα pathway, promoting cell survival. Inhibition of this pathway using BAY 11-7085 suppressed NF-κB activation and enhanced Paclitaxel-induced cytotoxicity, suggesting that targeting NF-κB signaling augmented the therapeutic efficacy of Paclitaxel in EOC [69]​.

### JAK/STAT pathway

The JAK/STAT pathway is instrumental in OC progression by facilitating cell proliferation, survival, invasion, metastasis, CSC maintenance, and chemoresistance. STAT proteins are aberrantly activated in EOC, positioning them as promising targets for therapeutic development. STAT1 and STAT3 are constitutively activated in EOCs resistant to chemotherapy and PARP inhibitors, with STAT1 overexpression prominently linked to Platinum resistance [70, 71]​. Current research provides limited insight into the role of STAT2, STAT4, STAT5, and STAT6 in EOC chemoresistance. However, one study highlighted the role of Fibrillin-1 (FBN1) overexpression in promoting Cisplatin resistance via the FBN1/VEGFR2/STAT2 axis by modulating glycolysis and angiogenesis [72]. Another study implicated STAT5 in mediating Carboplatin resistance in EOC with RelA. The inhibition of RelA and STAT5B using small molecule inhibitors decreased Bcl-xL expression and significantly increased Carboplatin sensitivity [63]​.

STAT3 is overexpressed in approximately 70% of HGSOC cases, correlating with poor overall survival. Recurrent ovarian tumors exhibited significantly higher levels of STAT3 than primary tumors. STAT3 activation drives anchorage-independent EOC cell growth, enabling survival in ascitic spheroids and contributing to chemoresistance [73]​. Paclitaxel and Cisplatin-resistant EOC cells also overexpressed pSTAT3 [74, 75]​. Beyond chemoresistance, STAT3 has also been implicated in radiotherapy resistance [76] ​. Growth hormones, cytokines from the IL-6 family, IL-10, IL-22, EGF, VEGF, TNFα, and prolactin activate STAT3, promoting cell proliferation, migration, and chemoresistance [77]. Inhibition of STAT3 reversed chemoresistance and enhanced chemotherapy-induced apoptosis, accompanied by decreased levels of the pro-survival genes Bcl-xL, Bcl-2, and survivin [78, 79]​.

STAT3 is also implicated in inducing stemness and Paclitaxel resistance in HGSOC via *L1CAM* [80]​. Decreased *Wip1* expression in EOC correlated with tumor metastasis and Platinum resistance mediated by a VEGF/STAT3-dependent mechanism [81]​. Increased STAT3 activation and upregulation of associated target genes (*cyclin D1*,* VEGF*,* Bcl-xL*) were observed in PARP inhibitor (Olaparib)-resistant EOC [82]​. STAT3 activation upregulated the oncogenic miRNA miR-216a, which suppressed PTEN, further driving tumor growth and Cisplatin resistance [83].

STAT3 and p53/RAS crosstalk has been reported to induce Cisplatin resistance through Slug/PI3K/MAPK/AKT-mediated EMT and autophagy [84]​. Cancer-associated fibroblasts induced resistance to Paclitaxel by promoting EMT via IL-6/JAK2/STAT3 axis activation [85]​. EMT regulator *Twist1* was also upregulated in Cisplatin-resistant EOC through STAT3, L1CAM, GAS6, and AKT activation [86]​. The crosstalk between the JAK/STAT3 and EGFR/ERK1/2 signaling cascades further contributes to chemoresistance. Combining the JAK2 inhibitor tyrphostin AG490 with the EGFR inhibitor gefitinib successfully reversed Cisplatin resistance [87]​.

### Notch pathway

The Notch pathway is frequently deregulated in EOC and is associated with advanced FIGO stages, chemoresistance, and reduced overall survival. The Notch-VEGF crosstalk regulates key cells in sprout formation, which plays a crucial role in angiogenesis [88]​. Notch knockdown downregulated the expression of *ABCB1* and *ABCC1*, resulting in increased Platinum chemosensitivity [89]. Additionally, the downregulation of Notch led to increased apoptosis, thereby reversing Cisplatin and Paclitaxel resistance [90].The use of a γ-secretase inhibitor to suppress the Notch pathway effectively reversed Platinum resistance [91]​. Additionally, the downregulation of Jagged1, a Notch ligand, increased sensitivity to docetaxel [92]​.

Notch1 intracellular domain (NICD) expression has been identified as an independent risk factor, correlating with advanced cancer stages and poor survival rates [93].Overexpression of Notch3 was observed in patients with recurrent HGSOC and associated with poor survival [94]​. In Notch3 overexpressing EOC cell lines, upregulation of CSC markers (*NANOG*,* OCT4*, and *SOX2*) and *ABCB1* contributed to Carboplatin chemoresistance [37]. Notch3 was also found to be overexpressed in ALDH1 + cell lines, rendering them resistant to Platinum compounds [95]​. Overexpression of *SUSD2*, a downstream target of Notch3, was associated with increased EMT and Cisplatin chemoresistance in HGSOC [96]​. Notch3 overexpression was correlated with reduced survival in patients with advanced EOC undergoing Platinum and Taxane therapy [97]​. Additionally, DLL4 was found to be overexpressed in 72% of EOC cases and identified as an independent prognostic marker of poor survival outcomes [98]​.

Treatment with Navicixizumab, an anti-DLL4/VEGF antibody, yielded encouraging results in Platinum-resistant EOC patients subjected to extensive prior treatment [99]​. Additionally, combination treatment with Demcizumab and Paclitaxel in heavily pre-treated, Platinum-resistant cases demonstrated an acceptable toxicity profile, warranting further investigation [100]​.

### TGF-β pathway

TGF-β exhibits a biphasic role in cancer progression, suppressing tumors in the early stages and promoting tumor growth in advanced stages [101]. In normal ovarian tissue, TGF-β signaling suppresses cellular growth and promotes differentiation. However, loss of this cytostatic activity is observed in almost 40% of OC cases [102]​. The loss of responsiveness is linked to downregulation of expression of TGF-β-associated genes and downstream signaling components [103]​. Beyond losing its cytostatic function, TGF-β remodels the TME, especially the extracellular matrix (ECM), contributing to therapy resistance [104]​.

Aberrant activation of the TGF-β pathway triggers anti-apoptotic mechanisms and chemoresistance. Experimental activation of TGF-β signaling has been utilized to induce Cisplatin resistance in the SKOV3 cell line [105]​. The pathway activates canonical EMT-related molecules, promoting Platinum resistance. Furthermore, TGF-β-mediated immunosuppression within the TME further contributes to chemoresistance [106]​. No clinically approved therapies target TGF-β, either as monotherapy or in combination, for treating HGSOC.

LY2109761 (Galunisertib), a small-molecule inhibitor targeting TGF-β receptors I/II, reduced drug resistance when combined with Cisplatin [107]​. *ZEB1*, a downstream target of TGF-β, promoted EMT and stemness by upregulating SOX2, OCT4, NANOG, CD44, and CD117, resulting in Cisplatin resistance [108]​. TGF-β/SMAD signaling upregulated *OLA1* and *ST3GAL1*, promoting EMT, increasing CSC features, and enhancing anti-apoptotic protein expression, including cleaved caspase-3, Bax, and Bcl-2 [109]​. *TGF-β1* knockdown was also reported to increase Cisplatin chemosensitivity and inhibit tumor growth by promoting BRCA1/Smad3 signaling in vitro [110]​.

Four commonly used chemotherapeutic agents for gynecological malignancies, Cisplatin, Paclitaxel, Doxorubicin, and Camptothecin, were found to activate TGF-β signaling via Smad2 and Smad3 phosphorylation. The subsequent stimulation of TGF-β1 production resulted in EMT, CSC enrichment, and decreased chemosensitivity. Combination treatment of trivalent TGF-β receptor trap, RER, with Cisplatin inhibited Cisplatin-induced TGF-β1 production and Cisplatin resistance, and effectively inhibited EOC growth [111]​.

Endoglin (ENG), a co-receptor in the canonical TGF-β pathway, promotes angiogenesis and chemoresistance in EOC. ENG was strongly expressed in both the tumor cells and vascular endothelial cells [112]​. ENG is a known marker of angiogenesis whose expression is activated in sprouting endothelial cells, establishing its relevance as a promising antiangiogenic target, even in cases where anti-VEGF therapies have failed [113]​. ENG expression was also associated with advanced disease stage, poor differentiation, chemoresistance, and a high recurrence rate [114]​. Treatment with Carotuximab (TRC105), an anti-ENG antibody, inhibited metastatic spread and improved overall survival in HGSOC in vivo [115]. Clinical trials reported reduced cancer antigen 125 (CA-125) levels in some patients treated with TRC105 [116]​. Furthermore, ENG was overexpressed in recurrent EOCs alongside Gli2, making it a promising target for overcoming chemoresistance [36]​.

### MAPK pathway

The RAS/MAPK pathway orchestrates chemoresistance in EOC by regulating key cellular functions such as proliferation, survival, and apoptosis, which are often dysregulated in cancer [117]​. Gain-of-function mutations in components of this pathway contribute to the constitutive activation of MAPK signaling, enabling EOC cells to thrive despite the presence of chemotherapeutic agents. As a result, patients harboring such aberrations frequently experience poor responses to standard Platinum-based chemotherapy [118]​. Combining MAPK inhibitors with conventional chemotherapeutic agents has the potential to enhance clinical efficacy and overcome drug resistance. Targeting the dysregulated MAPK signaling could help reverse resistance mechanisms and enable personalized therapeutic strategies, thereby improving outcomes in patients with chemoresistant EOC [119]​.

Activating mutations in the MAPK pathway are common in LGSOC [120]​. Increased activation of RAS and ERK1/2 is often observed in chemoresistant EOC [121]​. The RAS/MAPK signaling pathway, known for its pro-proliferative role, is activated in Platinum-resistant HGSOC [122]​. The PI3K/AKT and MAPK/RAS pathways together phosphorylate the pro-apoptotic protein BAD, inhibiting apoptosis and enhancing Platinum resistance [123]​. Inhibition of ERK1/2 reversed Paclitaxel-resistance, and the inhibition of Cisplatin-induced ERK1/2 activation increased Cisplatin sensitivity in both Cisplatin-resistant and Cisplatin-sensitive cells [124]​.

MEK phosphorylation activates mutant p53, driving Cisplatin resistance in HGSOC [125]​. Inadequate caspase-3 processing and MEKK1 activation induced a Cisplatin-resistant phenotype [126]​. Combining MEK inhibitors with PARP inhibitors reduced acquired resistance to PARP inhibitors and increased cytotoxicity synergistically. Combinations of Trametinib with Talazoparib and Pimasertib with Olaparib showed promising efficacy in reducing PARP inhibitor resistance [127, 128]​. Treatment of LGSOC patients with MEK inhibitors in combination with anti-hormonal therapy increased response rates and helped overcome therapy resistance [129]​. Combining MEK inhibitors with Paclitaxel demonstrated encouraging outcomes in Platinum-resistant EOC, where MAPK pathway alterations enhanced Paclitaxel-induced apoptosis [130]​.

Mitogen-activated protein kinase phosphatase-1 (MKP-1) expression enhanced Cisplatin resistance in EOC cells by stabilizing poly (ADP-ribose) polymerase-1 (PARP-1) levels through suppression of JNK1/2-mediated PARP-1 ubiquitination [131]​. ERK2 or MKP-1 downregulation enhanced Cisplatin-induced apoptosis by lowering Bcl-2 levels, suggesting that modulating the ERK-MKP-1-Bcl-2 axis or inhibiting PARP could overcome Cisplatin resistance [132]​. Downregulation of tumor suppressor long non-coding RNA *FER1L4* is linked to Paclitaxel resistance. Treatment with SB203580, a p38 MAPK inhibitor, increased FER1L4 levels and Paclitaxel sensitivity [133]​.

A Phase I CT (NCT05691504) is evaluating the safety of Pelcitoclax (APG-1252) and Cobimetinib, a reversible MEK1/2 inhibitor, in recurrent ovarian and endometrial cancer. Intermittent administration of dual RAF/MEK inhibitor VS-6766 in combination with FAK inhibitor Defactinib showed encouraging results in recurrent LGSOC patients in a Phase I CT (NCT03875820). ONC201, a dual AKT and ERK inhibitor, is currently under investigation in combination with Paclitaxel for treating Platinum-resistant EOC in an ongoing phase II CT (NCT04055649).

### Wnt/β-catenin pathway

The Wnt signaling pathway is tightly regulated, and its aberrant activity is implicated in numerous pathologies, including cancer. In EOC, Wnt signaling promotes proliferation, metastasis, chemoresistance, and stemness [134]​. Recent studies link Wnt signaling to immune evasion and tumor angiogenesis [135]​. The hypoxic TME upregulates CD117 in CSCs, leading to AKT-mediated phosphorylation of GSK3β and β-catenin nuclear accumulation. This increased nuclear localization of β-catenin enhances the expression of ABCG2, reducing sensitivity to Cisplatin and Paclitaxel [35]​. Furthermore, heightened nuclear activity of β-catenin is associated with increased chemoresistance in stem-like EOC cells [136]​.

Elevated LGR6 and LGR5 levels, markers of fallopian and ovarian CSCs, reflect heightened signaling of Wnt/β-catenin in HGSOC [137]​. Overexpression of R-spondin 1 (RSPO1), which binds to LGR4, LGR5, and LGR6 and contributes to the dysregulation of Wnt signaling, was found to enhance proliferation, migration, and chemoresistance in EOC [138, 139]​.

Canonical Wnt signaling pathway activation has been linked to reduced sensitivity to PARP inhibitors, particularly Olaparib, in HGSOC. Combining Wnt and PARP inhibitors may overcome acquired resistance in HGSOC [140]​. In the noncanonical Wnt pathway, Wnt5A is overexpressed in ovarian tumors and detected in ascites. Overexpression of Wnt5A receptor, receptor tyrosine kinase-like orphan receptor 1 (ROR1), is associated with reduced progression-free and overall survival in EOC [141]​. ROR1-positive xenograft cells exhibit stemness, marked by ALDH1 expression, spheroid formation, and tumorigenicity. These findings suggest that ROR1 may serve as a potential CSC marker and that noncanonical Wnt signaling contributes to EOC stemness and chemoresistance [142]​.

In a Phase Ib CT, Ipafricept, a Wnt inhibitor, showed tolerability with Carboplatin and Paclitaxel for recurrent Platinum-sensitive OC, though bone toxicity at effective doses limited further development [143]​.

### GAS6/AXL pathway

Growth arrest-specific 6 (GAS6), a receptor tyrosine kinase AXL ligand, has been identified as a potential target against EOC for its diverse role in tumor progression. The levels of *AXL* mRNA were high in several EOC cell lines, with GAS6 and AXL overexpression correlating with poor prognosis [144, 145]​. EOCs frequently have elevated *GAS6* mRNA and protein levels. Higher levels of *GAS6* mRNA were observed in patients with residual disease following initial cytoreductive surgery as compared to patients without residual tumor mass [146]​. Patients with higher expression of GAS6 in ovarian tumors failed to respond effectively to neoadjuvant chemotherapy, had poor progression-free survival and overall survival as compared to those with lower GAS6 expression levels. AVB-500, a GAS6/AXL inhibitor, received fast-track designation from the US FDA for the treatment of Platinum-resistant EOC [147]​. Combined treatment of AVB-500 with Carboplatin or Olaparib slowed replication fork progression and enhanced DNA damage, resulting in rapid death of EOC cells [147]​. Inhibition of AXL using a chemically modified aptamer prevented tumor growth and intraperitoneal EOC metastasis [144]​.

GAS6/AXL drives drug resistance by modulating the TME and crosstalk with PI3K, JAK/STAT, and MAPK pathways [148]​. AXL-related EMT is known to confer resistance to chemotherapy and targeted therapy [149]​. AXL activation by GAS6 inhibited apoptosis by activating PI3K/AKT and Ras/ERK pathway [150]​. MYD1, an AXL decoy receptor, inhibited GAS6-mediated phosphorylation of AXL, AKT, and ERK and suppressed peritoneal metastasis in EOC models [151]​. AXL inhibitors, including Bemcentinib and MYDI-72, increased sensitivity to Platinum, PARP, and ATR inhibitors by enhancing DNA damage [152]​. AXL inhibition also enhanced the sensitivity to chemotherapy and PARP inhibitors by increasing replication stress and DNA damage [147]​. GAS6/AXL also modulates MHC, PD-L1, and immunosuppressive chemokines, fostering an immunosuppressive TME [153]​.

Increased expression of AXL has also been associated with poor response to Carboplatin and Paclitaxel. Inhibition of AXL by BGB324 improved Carboplatin and Paclitaxel sensitivity in a dose-dependent manner in vitro [154]​. *AXL* mRNA was also significantly higher in Cisplatin-resistant cancer cells than in Cisplatin-sensitive cancer cells [155]​. GAS6 levels help in predicting the response to Platinum-based chemotherapeutics. Inhibition of the GAS6/AXL axis using AVB500 induce BRCAness (defect in homologous recombination repair mimicking BRCA1/2 loss) in sporadic HGSOC models by impairing homologous recombination repair, thereby enhancing sensitivity to Carboplatin [156]​. AXL inhibition also decreased phosphorylation of the M2 isoform of pyruvate kinase (PKM2), which in turn decreased glycolysis and increased Cisplatin sensitivity in EOC [157]​.

A Phase Ib/II CT (NCT03639246) of AVB-S6-500 (Batiraxcept) in combination with Paclitaxel or pegylated liposomal doxorubicin reported that the combination was well-tolerated and contributed to clinical efficacy of Paclitaxel in Platinum-resistant OC patients. Combination of Batiraxcept and Durvalumab in recurrent Platinum-resistant OC patients was well-tolerated but failed to demonstrate anti-tumor activity (NCT04019288) [158]. Tilvestamab, a humanized monoclonal antibody targeting AXL, which inhibits GAS6-driven activation of the AXL receptor, has been evaluated in patients with Platinum-resistant HGSOC as part of a Phase I CT (NCT04893551), although the results have not yet been reported.

### Hedgehog pathway

The Hedgehog (Hh) signaling pathway, a key modulator of embryonic development, is aberrantly activated in EOC, driving tumor growth, metastasis, and chemoresistance. The Hh signaling pathway has also been reported to regulate the growth of EOC spheroid forming cells, which exhibit CSC-like characteristics including self-renewal, differentiation, and chemoresistance [159]​. Inhibition of Hh signaling using cyclopamine decreased cell growth and induced apoptosis and G1 cell cycle arrest in ovarian carcinoma cells [160]​.

Overexpression of Gli1 and PTCH correlates with poor prognosis and overall survival in EOC. Ectopic overexpression of Gli1 enhances proliferation, motility, and invasiveness, acting as an independent prognostic marker [161]​. Gli1 modulates DNA adduct repair and influences Cisplatin sensitivity. Both Gli1 and SMO were overexpressed in Platinum-resistant EOC [162]​. Specifically, Gli1 overexpression increased phosphorylation of c-jun at Ser 63/73, subsequently enhancing *ERCC1* expression. Elevated ERCC1 levels contributed to Cisplatin resistance by reducing Platinum-induced DNA adduct formation [163]​. Studies revealed that solely the 130 kDa isoform of Gli1 could regulate the function of c-jun by binding to its promoter region. The elevation in the 130 kDa isoform of Gli1 was observed only in cancer cells, specifically Cisplatin-resistant EOC cells, and not normal ovarian cells [164]​.

SMO, Gli1, and Gli2 overexpression induced Paclitaxel resistance by upregulating ABCB1. SMO inhibition by Sonidegib restored Paclitaxel sensitivity but not Carboplatin sensitivity [165]​. Phase I CT reported the anti-tumor efficacy of Sonidegib with weekly Paclitaxel in patients with advanced solid tumors, including OC [166]​. The Hh signaling pathway, through its regulation of *ABCB1* as a downstream target of Gli2, induced Cisplatin resistance in EOC, and its inhibition enhanced Cisplatin sensitivity [167]​. Another study demonstrated that Gli1 upregulation directly elevated *ABCB1* and *ABCG2* expression and resulted in pump-enhanced drug resistance in spheroid forming cells [168].

## Role of MiRNAs in EOC chemoresistance

miRNAs are small (20–25 nucleotides), non-coding RNAs that regulate gene expression post-transcriptionally by targeting mRNAs for degradation or translational repression. They regulate approximately 30% of genes in the human genomes mainly by inhibiting gene expression. Owing to their ability to modulate a wide array of genes simultaneously, miRNAs serve as potent modulators of diverse cellular processes, including proliferation, apoptosis, differentiation, and stress responses [169]​. Their dysregulation has been extensively implicated in cancer pathogenesis, where they function either as oncogenes (onco-miRs) or tumor suppressors depending on the nature of their downstream targets [170]. This unique regulatory capacity presents miRNAs with significant therapeutic potential, as both biomarkers for diagnosis, prognosis, and drug response prediction, and as direct therapeutic targets [171]​.

In cancer, including EOC, miRNAs are often aberrantly expressed and contribute significantly to tumor progression, metastasis, and chemoresistance [172–174]​. Several miRNAs are differentially expressed between chemosensitive and chemoresistant EOC tissues and cell lines, and their functional relevance is increasingly recognized. Importantly, dysregulation of miRNAs contributes to chemoresistance through multiple, interconnected mechanisms, which include (i) suppression of apoptosis (e.g., miR-214, miR-199a, miR-21, miR-145), (ii) enhancement of DDR (e.g., miR-21, miR-140), (iii) activation of drug efflux transporters (e.g., miR-27a, miR-411, miR-1307), and (iv) promotion of EMT (e.g., miR-506, miR-181a, miR-363) and cancer stemness (e.g., miR-136) [175]​. These multifaceted roles make miRNAs central regulators of the resistant phenotype [176]​.

**Fig. 3:**
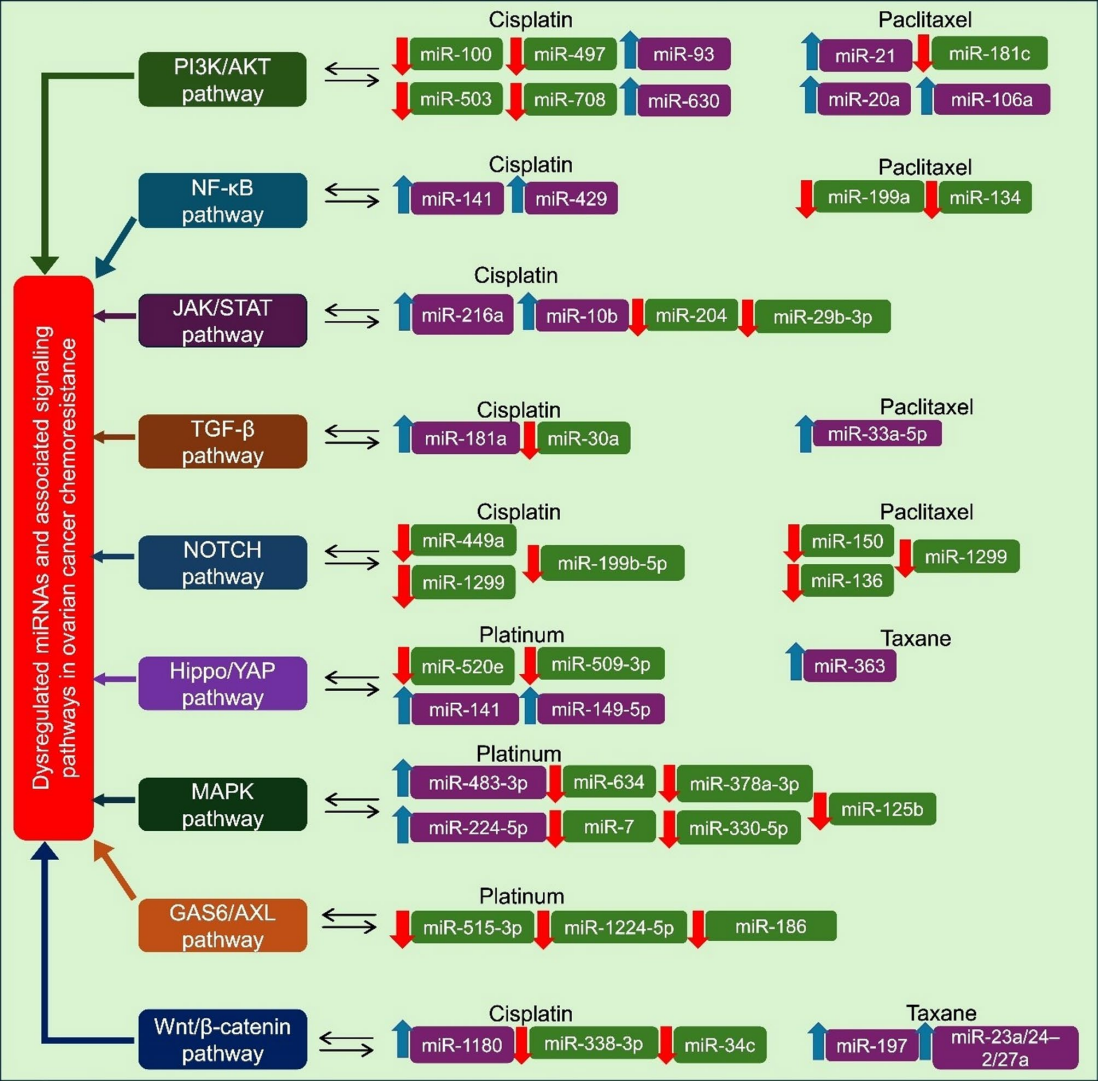
Dysregulated signaling pathways and associated miRNAs contributing to chemoresistance in EOC. Specific miRNAs that are upregulated (↑, blue) or downregulated (↓, red) in Platinum or Taxane resistant EOC are mapped alongside each pathway. These miRNAs modulate pathway activity, thereby influencing therapeutic resistance, and represent potential targets for overcoming drug resistance

Furthermore, miRNAs do not act in isolation but are intricately linked with major oncogenic signaling pathways [11]​. They form reciprocal feedback loops with pathways such as PI3K/AKT, Wnt/β-catenin, MAPK/ERK, STAT3, and TGF-β and can directly target upstream or downstream effectors in these pathways, thereby modulating their activity. Simultaneously, these pathways can transcriptionally regulate miRNA expression via specific transcription factors like c-Myc, p53, and NF-κB, establishing self-reinforcing regulatory circuits that maintain a chemoresistant phenotype (Fig. 3). This bidirectional crosstalk between miRNAs and signaling pathways has profound implications. The miR-200 family and ZEB1/2 form a double-negative feedback loop, regulating EMT and chemoresistance. Overexpression of miR-200 suppressed ZEB-mediated transcriptional repression, maintaining epithelial traits, while ZEB overexpression downregulated miR-200, promoting mesenchymal transition and drug resistance, highlighting this axis as a potential therapeutic target [177]​. Understanding this crosstalk offers insights into the molecular mechanisms of chemoresistance and informs the development of novel therapeutic strategies to overcome chemoresistance (Tables 2 and 3).

**Table 2:** Downregulated MiRNAs in chemoresistant EOC

miRNA	Associated pathway	Mechanism of action	Role in chemoresistance	Therapeutic modulators	References
miR-337-3p	PI3K/AKT	Targets PIK3CA and PIK3CB, suppresses PI3K/AKT signaling	Reverses chemoresistance and reduces proliferation	miR-337-3p mimic (in vitro); agomiR-337-3p (intratumoral, in vivo)	[203]
miR-199a-3p	PI3K/AKT	Inhibits mTOR	Reduces Cisplatin resistance and inhibits proliferation	miR-199a-3p mimic (in vitro); stable overexpression (orthotopic, in vivo); ITGB8 cDNA overexpression reverses miR-199a-3p function, WYE-354 (mTOR inhibitor)	​​ [204, 205]
miR-181c	PI3K/AKT	Downregulates GRP78, inhibits PI3K/AKT pathway	Reduces Paclitaxel resistance	miR-181c mimic (in vitro and intratumoral, in vivo); GRP78 siRNA mimics miR-181c effects	[206]
miR-100	PI3K/AKT	Downregulation of mTOR and PLK1	Promotes Cisplatin sensitivity	LV-miR-100 (in vitro and subcutaneous, in vivo)	[207]
miR-503	PI3K/AKT	Targets PI3K p85, suppress PI3K/AKT signaling	Enhances Cisplatin sensitivity	miR-503 mimic (in vitro); miR-503 agomir (intratumoral, in vivo)	[208]
miR-497	PI3K/AKT	Targets mTOR	Reduces Cisplatin resistance	miR-497 mimic (in vitro); Tet-On inducible miR-497 expression (in vivo)	[209]
miR-708	PI3K/AKT	Inhibition of AKT	Enhances Cisplatin sensitivity	miR-708 plasmid (in vitro)	[210]
miR-1271	PI3K/AKT	Downregulates mTOR expression	Enhances Cisplatin sensitivity	miR-1271 mimic/inhibitor (in vitro); simTOR co-transfection	[211]
miR-199a	NF-κB	Inhibits IKKβ, reduces NF-κB signaling	Reduces Paclitaxel resistance	None tested (expression only)	[212]
miR-134	NF-κB	Downregulated by NF-κB1, upregulated Table 1	Reduces Paclitaxel resistance	miR-134 mimic (in vitro)	[213]
miR-503-5p	NF-κB/JAK2/STAT3	Downregulated by NF-κB, inhibits CD97 mediated JAK2/STAT3 activation	Reduces Paclitaxel resistance	miR-503-5p mimic (in vitro)	[214]
miR-204	JAK/STAT	Targets IL-6R and STAT3	Reduces Cisplatin resistance	miR-204 mimic and IL-6R recombinant plasmid (in vitro and in vivo)	[215]
miR-29b-3p	JAK/STAT	Targets STAT3	Reduces carboplatin resistance	miR-29b-3p mimic and inhibitor (in vitro)	[216]
miR-136	Notch	Targets Notch3, inhibits NOTCH signaling	Reduces Paclitaxel resistance	miR-136 mimic (in vitro)	[217]
miR-199b-5p	Notch	Targets JAG1, attenuates JAG1/Notch1 signaling activity	Enhances Cisplatin-mediated cytotoxicity	miR-199b-5p mimic (plasmid, in vitro/in vivo); 5-Aza-dC (epigenetic reactivation)	[218]
miR-449a	Notch	Directly suppresses Notch1	Enhances Cisplatin sensitivity	miR-449a mimic (in vitro/intratumoral, in vivo); Notch1 siRNA (functional validation)	[89]
miR-150	Notch	Downregulates Notch3 and downstream proteins	Enhances Paclitaxel sensitivity	pre-miR-150 mimic (in vitro)	[219]
miR-1299	Notch	Negative regulation of Notch3 and NEK2	Enhances carboplatin and Paclitaxel sensitivity	miR-1299 mimic (in vitro); agomir-1299 (in vivo); circTNPO3 knockdown (shRNA, in vitro and in vivo)	​​ [220, 221]
miR-30a	TGF-β	Target SMAD4 to suppress autophagy activation	Reduces Cisplatin resistance	miR-30a mimic (in vitro); autophagy modulators (3-MA, rapamycin) as controls	[222]
miR-509-3p	Hippo/YAP	Targets YAP1, decrease Hippo signaling	Promotes Cisplatin sensitivity	miR-509-3p mimic (in vitro)	[223]
miR-520e	Hippo/YAP	Downregulates HLF and YAP, decreases Hippo signaling	Increases carboplatin sensitivity	miR-520e mimic (in vitro, in vivo); miR-520e sponge (in vitro)	[224]
miR-634	MAPK	Represses GRB2, ERK2, RSK2, inhibits Ras-MAPK pathway	Promotes sensitivity to Cisplatin, carboplatin and doxorubicin	miR-634 mimic and inhibitor (in vitro)	[225]
miR-125b	MAPK	Inhibits MKNK2, decreases MAPK signaling	Reduces chemoresistance	miR-125b mimic and inhibitor (in vitro)	[226]
miR-7	MAPK	Inhibits ERK	Reduces Cisplatin resistance	miR-7 mimic and inhibitor (in vitro and in vivo)	[227]
miR-378a-3p	MAPK	Negative regulation of MAPK1 and GRB2	Reduces Cisplatin resistance	miR-378a-3p mimic (in vitro)	[228]
miR-330-5p	MAPK	Regulates p38, JNK, and ERK levels by downregulating S100A7	Reduces Cisplatin resistance	miR-330-5p mimic (in vitro)	[229]
miR-515-3p	GAS6/AXL	Targets AXL	Promotes oxaliplatin sensitivity	miR-515-3p mimic (in vitro)	[230]
miR-1224-5p	GAS6/AKT	Downregulates SND1, suppresses GAS6/AKT signaling	Enhances Cisplatin sensitivity	miR-1224-5p mimic (in vitro)	[231, 232]
miR-186	GAS6/AKT	Reduces Twist1 expression, downregulation of GAS6/AKT pathway	Enhances Cisplatin sensitivity	miR-186 mimic (in vitro)	​​ [88, 233]
miR-338-3p	Wnt/β-catenin	Downregulates WNT2B	Enhances Cisplatin sensitivity	miR-338-3p mimic (in vitro, in vivo xenograft)	[234]
miR-34c	Wnt/β-catenin	Targets SOX9, suppresses β-catenin activity	Inhibits proliferation and Cisplatin chemoresistance	miR-34c mimic/inhibitor (in vitro)	[235]
miR-636	Hedgehog	Downregulates Gli2, inactivates Hedgehog pathway	Enhances Cisplatin sensitivity	miR-636 mimic, inhibitor (in vitro); agomiR-636 (in vivo)	[201, 236]

**Table 3:** Upregulated MiRNAs in chemoresistant EOC

miRNA	Associated pathway	Mechanism of action	Role in chemoresistance	Therapeutic modulators	References
miR-21	PI3K/AKT	Negative regulation of PTEN, enhance PI3K/AKT signaling	Promotes resistance to Cisplatin and Paclitaxel	miR-21 mimic/ inhibitor, HIF-1α siRNA (in vitro); ITX-0052 (SMI)	[237, 238]
miR-214	PI3K/AKT	Negative regulation of PTEN, enhance PI3K/AKT signaling	Promotes radio-resistance, inhibits Cisplatin-induced apoptosis, and enhances EOC cell progression	miR-214 mimic and inhibitor (in vitro)	[239–241]
miR-20a	PI3K/AKT	Negative regulation of PTEN, enhance PI3K/AKT signaling	Promotes Paclitaxel and Cisplatin resistance and EMT	sh-miR-20a (overexpression) and ASO-miR-20a (inhibitor) (in vitro)	[242, 243]
miR-221/222	PI3K/AKT	Negative regulation of PTEN, enhance PI3K/AKT signaling	Promotes Cisplatin resistance	FAM-miR-221/222 inhibitor (Lipofectamine-based) (in vitro)	[244]
miR-93	PI3K/AKT	Negative regulation of PTEN, enhance PI3K/AKT signaling	Promotes Cisplatin resistance	ASO-miR-93 (inhibition) (in vitro)	[245]
miR-630	PI3K/AKT	Negative regulation of PTEN, enhance PI3K/AKT signaling	Promotes Cisplatin resistance	miR-630 inhibitor (in vitro)	[246]
miR-205-5p	PI3K/AKT	Inhibition of PTEN/AKT pathway	Promotes Cisplatin resistance	miR-205-5p mimic and inhibitor (in vitro)	[247]
miR-106a	PI3K/AKT	Negative regulation of PTEN, enhance PI3K/AKT signaling	Promotes Paclitaxel resistance	miR-106a inhibitor (in vitro), Tocilizumab (anti-IL6R mAb) indirect downregulation of miR-106a	[248, 249]
miR-223	PI3K/AKT	Negative regulation of PTEN, enhance PI3K/AKT signaling	Promotes Cisplatin resistance	miR-223 agomir/antagomir (in vivo)	[250]
miR-141	NF-κB	Regulates KEAP1, activate NF-κB pathway	Promotes Cisplatin resistance	miR-141 mimic and inhibitor, KEAP1 siRNA (in vitro)	[251]
miR-429	NF-κB	Upregulated by NF-κB, downregulated CASR and upregulated STAT3	Promotes Cisplatin resistance	miR-429 mimic and inhibitor, CASR siRNA (in vitro); miR-429 agomir/antagomir (in vivo)	[252]
miR-452-5p, miR-105-5p	NF-κB	Regulated by RelA and RelB, activate NF-κB pathway	Promotes chemoresistance in stem-like tumor initiating cells	miR-452-5p mimic and inhibitor; miR-335-5p mimic and inhibitor; inducible shRelA/shRelB (doxycycline, in vitro)	[253]
miR-216a	JAK/STAT	Upregulated by STAT3 overexpression, downregulates PTEN	Promotes Cisplatin resistance	miR-216a mimic and inhibitor, PTEN overexpression (in vitro)	[254]
miR-10b	JAK/STAT	Activates STAT3	Promotes Cisplatin resistance	miR-10b mimic (in vitro); CHRF GapmeR (lncRNA silencing, in vitro)	[255]
miR-181a	TGF-β	Targets SMAD7, modulates TGF-β-mediated EMT	Promotes Platinum resistance	miR-181a retroviral overexpression, miR-181a sensor & decoy vectors (in vitro)	[256]
miR-33a-5p	TGF-β	Downregulates CROT expression, increased nuclear localization of Smad2/4,	Promotes Paclitaxel resistance	miR-33a-5p mimic and inhibitor; CROT siRNA/plasmid (in vitro)	[257]
miR-141	Hippo/YAP	Targets YAP1, activates YAP1/GROα/CXCR1/2 signaling cascade	Promotes Cisplatin resistance	miR-141 mimic (overexpression) (in vitro)	[258]
miR-363	Hippo/YAP	Decreases LATS2 leading to increased YAP nuclear localization	Promotes Taxane resistance	pCMV-miR-363 (expression vector), miR-363 inhibitor	[259]
miR-149-5p	Hippo/YAP	Increased nuclear localization of YAP and TAZ	Promotes Cisplatin resistance	pMSCV-miR-149-5p and anti-miR-149-5p (in vitro)	[260]
miR-483-3p	MAPK	Inhibition of PRKCA	Promotes Cisplatin resistance	Pre-miR-483-3p mimic and miR-483-3p inhibitor (in vitro)	​​ [261]
miR-224-5p	MAPK	Downregulation of PRKCD	Promotes Platinum resistance	miR-224-5p mimic and inhibitor (in vitro)	[262]
miR-197	Wnt/β-catenin	Downregulate NLK, activate Wnt signaling	Promotes taxol resistance	miR-197 mimic and inhibitor (in vitro)	[263]
miR-1180	Wnt/β-catenin	Targets SFRP1, enhances Wnt signaling	Promotes Cisplatin resistance	miR-1180 mimic and anti-miR-1180 (in vitro); BM-MSC-derived exosomes (ex vivo, in vitro)	​​ [264]
miR-92a	Wnt/β-catenin	Targets DKK1, increases β-catenin activity	Promotes stemness and chemoresistance	STAT3 knockdown mediated miR-92a suppression (in vitro, in vivo); doxycycline-inducible STAT3 shRNA	[73]

Therapeutic modulation of miRNAs through synthetic mimics to restore tumor suppressor miRNAs or antago-miRs to inhibit onco-miRs, has shown promise in several preclinical models. Despite these advances, several obstacles impede the clinical translation of miRNA-based therapies, including the need for efficient and tumor-specific delivery systems, ensuring molecular stability in circulation, and minimizing off-target effects that could lead to unintended gene regulation or toxicity [178]​. Ongoing developments in nanoparticle-based delivery systems and miRNA sponges have addressed these limitations to a certain extent. The integration of miRNA-based diagnostics and therapeutics into clinical practice holds the potential to overcome the current limitations of chemoresistance in EOC.

## Conclusion

Chemoresistance remains the principal barrier to effective and durable therapy in EOC. This phenotype is driven by a complex interplay of signaling pathways that promote cellular survival, suppress apoptosis, induce EMT, and remodel the TME to support immune evasion and drug efflux. miRNAs act as both regulators and effectors within these circuits, modulating multiple resistance-associated processes such as DDR, stemness, and survival via tightly regulated feedback loops.

Among these, the PI3K/AKT/mTOR axis acts as a central signaling hub, coordinating pro-survival cues and interacting with other pathways and miRNAs. These insights highlight miRNAs not only as regulators but also as potential diagnostic and prognostic biomarkers. Targeting key nodes within these miRNA–signaling networks represents a promising strategy to overcome resistance. However, therapeutic progress is challenged by tumor heterogeneity and the limited bioavailability of RNA-based interventions. Bridging these gaps requires mechanistic precision and better translational frameworks.

## Future perspectives

Tackling chemoresistance in EOC demands a focused effort to unravel how miRNAs interact with key signaling pathways in a subtype and stage-specific manner, laying the groundwork for more precise and durable interventions. Identifying resistance-driving miRNA signatures through spatial transcriptomics, single-cell profiling, and multi-omics could reveal functional heterogeneity and resistant niches within tumors. Functional CRISPR/Cas9 screening may uncover previously unrecognized, targetable feedback loops.

Therapeutically, miRNA-based agents capable of modulating multiple signaling networks show significant potential, especially when combined with existing chemotherapeutic agents or specific pathway inhibitors. However, issues of delivery, stability, and specificity remain a challenge. These limitations could be addressed by nanotechnology-based formulations such as lipid nanoparticles, tumor-targeting exosomes, or peptide-conjugated carriers. Integrating Artificial Intelligence/ Machine Learning models to predict resistance trajectories and optimize personalized drug sequencing will be beneficial [179]. Exploring systems-level therapeutic strategies may offer promising avenues to restore drug sensitivity, limit relapse, and improve long-term outcomes in chemoresistant EOC.

## Data Availability

No datasets were generated or analysed during the current study.

## Abbreviations

ABC: ATP-Binding Cassette
AIF: Apoptosis-Initiating Factor
CA-125: Cancer Antigen 125
cIAP1: Cellular Inhibitors of Apoptosis Protein-1
CSC: Cancer Stem Cell
DDR: DNA Damage Repair
DHMEQ: Dehydroxymethylepoxyquinomicin
DNA-PK: DNA-Dependent Protein Kinase
EMT: Epithelial-To-Mesenchymal Transition
ENG: Endoglin
EOC: Epithelial Ovarian Cancer
FBN1: Fibrillin-1
GAS6: Growth Arrest-Specific 6
HGSOC: High-Grade Serous Ovarian Carcinoma
Hh: Hedgehog
IAPs: Inhibitors of Apoptosis
LGSOC: Low-Grade Serous Ovarian Carcinomas
MAPKs: MAP kinases
MAPs: Microtubule-Associated Proteins
MDR: Multidrug Resistance
MDSCs: Myeloid-Derived Suppressor Cells
MKP-1: Mitogen-Activated Protein Kinase Phosphatase-1
NICD: Notch1 Intracellular Domain
OC: Ovarian Cancer
PIK3CA: Phosphatidylinositol-4,5-Bisphosphate 3-Kinase Catalytic SubunitΑ
PKM2: M2 Isoform of Pyruvate Kinase
ROR1: Receptor Tyrosine Kinase-Like Orphan Receptor 1
RSPO1: R-Spondin 1
TME: Tumor Microenvironment
XIAPs: X-Linked IAPs

## References

[1] Luvero D, Plotti F, Aloisia A, Montera R, Terranova C, Cicco Nardone C. Ovarian cancer relapse: from the latest scientific evidence to the best practice. Crit Rev Oncol Hematol 140:28–38. 10.1016/j.critrevonc.2019.05.014 31176270

[2] Peres LC, Cushing-Haugen KL, Köbel M, Harris HR, Berchuck A, Rossing MA et al. Invasive Epithelial Ovarian Cancer Survival by Histotype and Disease Stage. JNCI J Natl Cancer Inst. 2019;111:60–8. Available from: https://academic.oup.com/jnci/article/111/1/60/4990606 10.1093/jnci/djy071 PMC6335112 29718305

[3] Baldwin L, Ware R, Huang B, Tucker T, Goodrich S, Podzielinski I. Ten-year relative survival for epithelial ovarian cancer. Gynecol Oncol. 120:S34–5. 10.1097/AOG.0b013e318264f794 22914471

[4] Torre LA, Trabert B, DeSantis CE, Miller KD, Samimi G, Runowicz CD, et al. Ovarian cancer statistics, 2018. CA Cancer J Clin. 2018;68:284–96. 29809280 10.3322/caac.21456 PMC6621554

[5] Koti M, Siu A, Clément I, Bidarimath M, Turashvili G, Edwards A, et al. A distinct pre-existing inflammatory tumour microenvironment is associated with chemotherapy resistance in high-grade serous epithelial ovarian cancer. Br J Cancer. 2015;112:1215–22. 25826225 10.1038/bjc.2015.81 PMC4385963

[6] Luvero D, Milani A, Ledermann JA. Treatment options in recurrent ovarian cancer: latest evidence and clinical potential. Ther Adv Med Oncol. 6:229–39. 10.1177/1758834014544121 PMC4206613 25342990

[7] Wang L, Wang X, Zhu X, Zhong L, Jiang Q, Wang Y, et al. Drug resistance in ovarian cancer: from mechanism to clinical trial. Mol Cancer. 2024;23:66. 38539161 10.1186/s12943-024-01967-3 PMC10976737

[8] Nunes M, Bartosch C, Abreu MH, Richardson A, Almeida R, Ricardo S. Deciphering the Molecular Mechanisms behind Drug Resistance in Ovarian Cancer to Unlock Efficient Treatment Options. Cells. 2024;13:786. Available from: https://www.mdpi.com/2073-4409/13/9/786 10.3390/cells13090786 PMC11083313 38727322

[9] You M, Xie Z, Zhang N, Zhang Y, Xiao D, Liu S, et al. Signaling pathways in cancer metabolism: mechanisms and therapeutic targets. Signal Transduct Target Ther. 2023;8:196. 37164974 10.1038/s41392-023-01442-3 PMC10172373

[10] Hu X-YX-Y, Song Z, Yang Z-W, Li J-JJ-J, Liu J, Wang. H-SH-S. Cancer drug resistance related micrornas: recent advances in detection methods. Analyst. 2022;147:2615–32. 35611577 10.1039/d2an00171c

[11] Shekhar R, Kumari S, Vergish S, Tripathi P. The crosstalk between miRNAs and signaling pathways in human cancers: Potential therapeutic implications. 2024. pp. 133–65. Available from: https://linkinghub.elsevier.com/retrieve/pii/S1937644823001715 10.1016/bs.ircmb.2023.12.001 38782498

[12] Wolde T, Bhardwaj V, Reyad-ul-Ferdous M, Qin P, Pandey V. The Integrated Bioinformatic Approach Reveals the Prognostic Significance of LRP1 Expression in Ovarian Cancer. Int J Mol Sci. 2024;25:7996. Available from: https://www.mdpi.com/1422-0067/25/14/7996 10.3390/ijms25147996 PMC11276689 39063239

[13] Bhardwaj V, Zhang X, Pandey V, Garg M. Neo-vascularization-based therapeutic perspectives in advanced ovarian cancer. Biochim Biophys Acta - Rev Cancer. 2023;1878:188888. Available from: https://linkinghub.elsevier.com/retrieve/pii/S0304419X23000379 10.1016/j.bbcan.2023.188888 37001618

[14] Bhardwaj V, Tan YQ, Wu MM, Ma L, Zhu T, Lobie PE et al. Long non-coding RNAs in recurrent ovarian cancer: Theranostic perspectives. Cancer Lett. 2021;502:97–107. Available from: https://linkinghub.elsevier.com/retrieve/pii/S0304383521000100 10.1016/j.canlet.2020.12.042 33429007

[15] Binju M, Amaya-Padilla MA, Wan G, Gunosewoyo H, Suryo Rahmanto Y, Yu Y. Therapeutic Inducers of Apoptosis in Ovarian Cancer. Available from: https://www.mdpi.com/2072-6694/11/11/1786 10.3390/cancers11111786 PMC6896143 31766284

[16] Mansouri A, Zhang Q, Ridgway LD, Tian L, Claret F-X. Cisplatin Resistance in an Ovarian Carcinoma Is Associated With a Defect in Programmed Cell Death Control Through XIAP Regulation. Oncol Res Featur Preclin Clin Cancer Ther. Available from: https://www.ingentaconnect.com/content/10.3727/096504003108748410 10.3727/096504003108748410 PMC4089892 12725530

[17] HABATA S, IWASAKI M, SUGIO A, SUZUKI M, TAMATE M. SATOHISA S. BAG3-mediated Mcl-1 stabilization contributes to drug resistance via interaction with USP9X in ovarian cancer. Int J Oncol. Available from: https://www.spandidos-publications.com/10.3892/ijo.2016.3494 10.3892/ijo.2016.3494 27120977

[18] Weng D, Song X, Xing H, Ma X, Xia X, Weng Y. Implication of the Akt2/survivin pathway as a critical target in paclitaxel treatment in human ovarian cancer cells. Cancer Lett. Available from: https://linkinghub.elsevier.com/retrieve/pii/S0304383508006319 10.1016/j.canlet.2008.08.027 18842333

[19] Ma Q, Liu Z, Wang T, Zhao P, Liu M, Wang Y. Resensitizing Paclitaxel-Resistant Ovarian Cancer via Targeting Lipid Metabolism Key Enzymes CPT1A, SCD and FASN. Int J Mol Sci. Available from: https://www.mdpi.com/1422-0067/24/22/16503 10.3390/ijms242216503 PMC10671839 38003694

[20] Pilié PG, Tang C, Mills GB, Yap TA. State-of-the-art strategies for targeting the DNA damage response in cancer. Nat Rev Clin Oncol. Available from: https://www.nature.com/articles/s41571-018-0114-z 10.1038/s41571-018-0114-z PMC8327299 30356138

[21] Damia G, Broggini M. Platinum Resistance in Ovarian Cancer: Role of DNA Repair. Available from: https://www.mdpi.com/2072-6694/11/1/119 10.3390/cancers11010119 PMC6357127 30669514

[22] Guffanti F, Fratelli M, Ganzinelli M, Bolis M, Ricci F, Bizzaro F. Platinum sensitivity and DNA repair in a recently established panel of patient-derived ovarian carcinoma xenografts. Oncotarget. Available from: https://www.oncotarget.com/lookup/doi/10.18632/oncotarget.25185 10.18632/oncotarget.25185 PMC5973859 29872499

[23] Liu X, Yu Y, Zhang J, Lu C, Wang L, Liu P. HDAC1 Silencing in Ovarian Cancer Enhances the Chemotherapy Response. Cell Physiol Biochem. Available from: https://karger.com/article/doi/10.1159/000492260 10.1159/000492260 30071534

[24] Maloney SM, Hoover CA, Prosperi VM-LL. JR. Mechanisms of Taxane Resistance. Available from: https://www.mdpi.com/2072-6694/12/11/3323 10.3390/cancers12113323 PMC7697134 33182737

[25] Du J, Li B, Fang Y, Liu Y, Wang Y, Li J. Overexpression of Class III β-tubulin, Sox2, and nuclear Survivin is predictive of taxane resistance in patients with stage III ovarian epithelial cancer. BMC Cancer. Available from: https://bmccancer.biomedcentral.com/articles/10.1186/s12885-015-1553-x 10.1186/s12885-015-1553-x PMC4511538 26198101

[26] Yu Y, Gaillard S, Phillip JM, Huang T-C, Pinto SM, Tessarollo NG. Inhibition of Spleen Tyrosine Kinase Potentiates Paclitaxel-Induced Cytotoxicity in Ovarian Cancer Cells by Stabilizing Microtubules. Cancer Cell. Available from: https://linkinghub.elsevier.com/retrieve/pii/S1535610815001853 10.1016/j.ccell.2015.05.009 PMC5257279 26096845

[27] Smoter M, Bodnar L, Grala B, Stec R, Zieniuk K, Kozlowski W. Tau protein as a potential predictive marker in epithelial ovarian cancer patients treated with paclitaxel/platinum first-line chemotherapy. J Exp Clin Cancer Res. Available from: https://jeccr.biomedcentral.com/articles/10.1186/1756-9966-32-25 10.1186/1756-9966-32-25 PMC3654950 23631819

[28] Raab M, Sanhaji M, Zhou S, Rödel F, El-Balat A, Becker S. Blocking Mitotic Exit of Ovarian Cancer Cells by Pharmaceutical Inhibition of the Anaphase-Promoting Complex Reduces Chromosomal Instability. Neoplasia. Available from: https://linkinghub.elsevier.com/retrieve/pii/S1476558619300351 10.1016/j.neo.2019.01.007 PMC6407080 30851646

[29] Ribatti D, Tamma R, Annese T. Epithelial-Mesenchymal Transition in Cancer: A Historical Overview. Transl Oncol. Available from: https://linkinghub.elsevier.com/retrieve/pii/S1936523320302503 10.1016/j.tranon.2020.100773 PMC7182759 32334405

[30] Loret N, Denys H, Tummers P, Berx G. The Role of Epithelial-to-Mesenchymal Plasticity in Ovarian Cancer Progression and Therapy Resistance. Available from: https://www.mdpi.com/2072-6694/11/6/838 10.3390/cancers11060838 PMC6628067 31213009

[31] Bonito NA, Borley J, Wilhelm-Benartzi CS, Ghaem-Maghami S, Brown R. Epigenetic Regulation of the Homeobox Gene MSX1 Associates with Platinum-Resistant Disease in High-Grade Serous Epithelial Ovarian Cancer. Clin Cancer Res. Available from: https://aacrjournals.org/clincancerres/article/22/12/3097/122035/Epigenetic-Regulation-of-the-Homeobox-Gene-MSX1 10.1158/1078-0432.CCR-15-1669 PMC4849558 26763252

[32] Bates M, Spillane CD, Gallagher MF, McCann A, Martin C, Blackshields G. The role of the MAD2-TLR4-MyD88 axis in paclitaxel resistance in ovarian cancer. In: Chan DW, editor. PLoS One. Available from: 10.1371/journal.pone.0243715 10.1371/journal.pone.0243715 PMC7769460 33370338

[33] Moisan F, Francisco EB, Brozovic A, Duran GE, Wang YC, Chaturvedi S. Enhancement of paclitaxel and carboplatin therapies by CCL2 blockade in ovarian cancers. Mol Oncol. Available from: https://febs.onlinelibrary.wiley.com/doi/10.1016/j.molonc.2014.03.016 10.1016/j.molonc.2014.03.016 PMC4801026 24816187

[34] Shi Y, Zhang J, Liu M, Huang Y, Yin L. SMAD3 inducing the transcription of STYK1 to promote the EMT process and improve the tolerance of ovarian carcinoma cells to paclitaxel. J Cell Biochem. Available from: https://onlinelibrary.wiley.com/doi/10.1002/jcb.28371 10.1002/jcb.28371 30701575

[35] Chau WK, Ip CK, Mak ASC, Lai H-C, Wong AST. c-Kit mediates chemoresistance and tumor-initiating capacity of ovarian cancer cells through activation of Wnt/β-catenin–ATP-binding cassette G2 signaling. Oncogene. Available from: https://www.nature.com/articles/onc2012290 10.1038/onc.2012.290 22797058

[36] Steg AD, Bevis KS, Katre AA, Ziebarth A, Dobbin ZC, Alvarez RD. Stem cell pathways contribute to clinical chemoresistance in ovarian cancer. Clin Cancer Res 18:869–81. 10.1158/1078-0432.CCR-11-2188 PMC3271164 22142828

[37] Park JT, Chen X, Tropè CG, Davidson B, Shih I-M, Wang T-L. Notch3 overexpression is related to the recurrence of ovarian cancer and confers resistance to carboplatin. Am J Pathol 177:1087–94. 10.2353/ajpath.2010.100316 PMC2928943 20671266

[38] Carden CP, Stewart A, Thavasu P, Kipps E, Pope L, Crespo M. The Association of PI3 Kinase Signaling and Chemoresistance in Advanced Ovarian Cancer. Available from: https://aacrjournals.org/mct/article/11/7/1609/91262/The-Association-of-PI3-Kinase-Signaling-and 10.1158/1535-7163.MCT-11-0996 PMC4630857 22556379

[39] Deng J, Bai X, Feng X, Ni J, Beretov J, Graham P. Inhibition of PI3K/Akt/mTOR signaling pathway alleviates ovarian cancer chemoresistance through reversing epithelial-mesenchymal transition and decreasing cancer stem cell marker expression. BMC Cancer. 19. 10.1186/s12885-019-5824-9 PMC6591840 31234823

[40] Marchetti C, Felice F, Romito A, Iacobelli V, Sassu CM, Corrado G. Chemotherapy resistance in epithelial ovarian cancer: mechanisms and emerging treatments. Semin Cancer Biol 77:144–66. 10.1016/j.semcancer.2021.08.011 34464704

[41] Fraser M, Leung BM, Yan X, Dan HC, Cheng JQ, Tsang BK. p53 is a determinant of X-linked inhibitor of apoptosis protein/Akt-mediated chemoresistance in human ovarian cancer cells. Cancer Res 63:7081–8. 14612499

[42] Cheng JQ, Jiang X, Fraser M, Li M, Dan HC, Sun M. Role of X-linked inhibitor of apoptosis protein in chemoresistance in ovarian cancer: possible involvement of the phosphoinositide-3 kinase/akt pathway. Drug Resist Updat 5:131–46. 10.1016/s1368-7646(02)00003-1 12237081

[43] Ghoneum A, Said N. PI3K-AKT-mTOR and NFκB pathways in ovarian cancer: implications for targeted therapeutics. Cancers (Basel). 10.3390/cancers11070949 PMC6679095 31284467

[44] Liu R, Chen Y, Liu G, Li C, Song Y, Cao Z. PI3K/AKT pathway as a key link modulates the multidrug resistance of cancers. Cell Death Dis. 11. 10.1038/s41419-020-02998-6 PMC7515865 32973135

[45] Ma S, Wang J, Cui Z, Yang X, Cui X, Li X. HIF-2α-dependent TGFBI promotes ovarian cancer chemoresistance by activating PI3K/Akt pathway to inhibit apoptosis and facilitate DNA repair process. Sci Rep. 10.1038/s41598-024-53854-y PMC10873328 38365849

[46] Wang Y, Chiou Y-S, Chong Q-Y, Zhang M, Rangappa KS, Ma L et al. Pharmacological Inhibition of BAD Ser99 Phosphorylation Enhances the Efficacy of Cisplatin in Ovarian Cancer by Inhibition of Cancer Stem Cell-like Behavior. ACS Pharmacol Transl Sci. 2020;3:1083–99. Available from: 10.1021/acsptsci.0c00064 10.1021/acsptsci.0c00064 PMC7737213 33344891

[47] Brasseur K, Gévry N, Asselin E. Chemoresistance and targeted therapies in ovarian and endometrial cancers. Oncotarget. 8:4008–42. 10.18632/oncotarget.14021 PMC5354810 28008141

[48] Stronach EA, Chen M, Maginn EN, Agarwal R, Mills GB, Wasan H. DNA-PK mediates AKT activation and apoptosis Inhibition in clinically acquired platinum resistance. Neoplasia 13. 10.1593/neo.111032 PMC3223610 22131882

[49] Wu Y-H, Huang Y-F, Chen C-C, Huang C-Y, Chou C-Y. Comparing PI3K/Akt inhibitors used in ovarian cancer treatment. Front Pharmacol. 11. 10.3389/fphar.2020.00206 PMC7063971 32194423

[50] Choi HJ, Heo JH, Park JY, Jeong JY, Cho HJ, Park KS. A novel PI3K/mTOR dual inhibitor, CMG002, overcomes the chemoresistance in ovarian cancer. Gynecol Oncol 153:135–48. 10.1016/j.ygyno.2019.01.012 30686552

[51] Tomek K, Wagner R, Varga F, Singer CF, Karlic H, Grunt TW. Blockade of fatty acid synthase induces ubiquitination and degradation of Phosphoinositide-3-Kinase signaling proteins in ovarian cancer. Mol Cancer Res 9:1767–79. 10.1158/1541-7786.MCR-10-0467 21970855

[52] Bauerschlag DO, Maass N, Leonhardt P, Verburg FA, Pecks U, Zeppernick F. Fatty acid synthase overexpression: target for therapy and reversal of chemoresistance in ovarian cancer. J Transl Med 13. 10.1186/s12967-015-0511-3 PMC4504229 25947066

[53] Shi Y, Meng X, Xu Y, Tian X. Role of < scp > foxo protein’s abnormal activation through < scp > PI3K / <scp > AKT pathway in platinum resistance of ovarian cancer</scp >. J Obstet Gynaecol Res 47:1946–57. 10.1111/jog.14753 33827148

[54] Wang J, Yang H, Li W, Xu H, Yang X, Gan L. Thioredoxin 1 upregulates FOXO1 transcriptional activity in drug resistance in ovarian cancer cells. Biochim Biophys Acta - Mol Basis Dis 1852:395–405. 10.1016/j.bbadis.2014.12.002 25483711

[55] Lin J, Song T, Li C, Mao W. GSK-3β in DNA repair, apoptosis, and resistance of chemotherapy, radiotherapy of cancer. Biochim Biophys Acta - Mol Cell Res 1867. 10.1016/j.bbamcr.2020.118659 31978503

[56] Mundi PS, Sachdev J, McCourt C, Kalinsky K. AKT in cancer: new molecular insights and advances in drug development. Br J Clin Pharmacol 82:943–56. 10.1111/bcp.13021 PMC5137819 27232857

[57] Gao C, Yuan X, Jiang Z, Gan D, Ding L, Sun Y et al. Regulation of AKT phosphorylation by GSK3$β$ and PTEN to control chemoresistance in breast cancer. Breast Cancer Res Treat. 2019;176:291–301. Available from: http://link.springer.com/10.1007/s10549-019-05239-3 10.1007/s10549-019-05239-3 31006103

[58] Yang G, Xiao X, Rosen DG, Cheng X, Wu X, Chang B. The biphasic role of NF-κB in progression and chemoresistance of ovarian cancer. Clin Cancer Res 17:2181–94. 10.1158/1078-0432.CCR-10-3265 PMC3152795 21339307

[59] Walz A, Ugolkov A, Chandra S, Kozikowski A, Carneiro BA, O ’Hallora. V. T. Molecular Pathways: Revisiting Glycogen Synthase Kinase-3β as a Target for the Treatment of Cancer. Clin Cancer Res. Available from: https://aacrjournals.org/clincancerres/article/23/8/1891/123079/Molecular-Pathways-Revisiting-Glycogen-Synthase 10.1158/1078-0432.CCR-15-2240 PMC5392367 28053024

[60] Shen H, Liao B, Wan Z, Zhao Y, You Z, Liu J. PTOV1 promotes cisplatin-induced chemotherapy resistance by activating the nuclear factor kappa B pathway in ovarian cancer. Mol Ther Oncolytics 20:499–507. 10.1016/j.omto.2021.02.008 PMC7937561 33738336

[61] Harrington BS, Annunziata CM. NF-κB Signaling in Ovarian Cancer. Available from: https://www.mdpi.com/2072-6694/11/8/1182 10.3390/cancers11081182 PMC6721592 31443240

[62] Nishio H, Yaguchi T, Sugiyama J, Sumimoto H, Umezawa K, Iwata T. Immunosuppression through constitutively activated NF-κB signalling in human ovarian cancer and its reversal by an NF-κB inhibitor. Br J Cancer. 110:2965–74. 10.1038/bjc.2014.251 PMC4056060 24867687

[63] Jinawath N, Vasoontara C, Jinawath A, Fang X, Zhao K, Yap K-L. Oncoproteomic analysis reveals Co-Upregulation of RELA and STAT5 in carboplatin resistant ovarian carcinoma. In: Cordes N, editor PLoS One. 10.1371/journal.pone.0011198 PMC2887843 20585448

[64] Michalak M, Lach MS, Borska S, Nowakowski B, Umezawa K, Suchorska WM. DHMEQ enhances the cytotoxic effect of cisplatin and carboplatin in ovarian cancer cell lines. Am J Cancer Res 11:6024–41. PMC8727817 35018240

[65] Harte MT, Gorski JJ, Savage KI, Purcell JW, Barros EM, Burn PM. NF-κB is a critical mediator of BRCA1-induced chemoresistance. Oncogene 33:713–23. 10.1038/onc.2013.10 PMC3917825 23435429

[66] Hoeflich KP, Luo J, Rubie EA, Tsao M-S, Jin O, Woodgett JR. Requirement for glycogen synthase kinase-3β in cell survival and NF-κB activation. Nat. Available from: https://www.nature.com/articles/35017574 10.1038/35017574 10894547

[67] Ougolkov AV, Bone ND, Fernandez-Zapico ME, Kay NE, Billadeau DD. Inhibition of glycogen synthase kinase-3 activity leads to epigenetic silencing of nuclear factor $κ$B target genes and induction of apoptosis in chronic lymphocytic leukemia B cells. Blood. 2007;110:735–42. Available from: https://ashpublications.org/blood/article/110/2/735/22903/Inhibition-of-glycogen-synthase-kinase3-activity 10.1182/blood-2006-12-060947 PMC1924475 17463171

[68] Mabuchi S, Ohmichi M, Nishio Y, Hayasaka T, Kimura A, Ohta T. Inhibition of NFκB Increases the Efficacy of Cisplatin in in Vitro and in Vivo Ovarian Cancer Models. J Biol Chem. Available from: https://linkinghub.elsevier.com/retrieve/pii/S0021925820667205 10.1074/jbc.M313709200 15026414

[69] Mabuchi S, Ohmichi M, Nishio Y, Hayasaka T, Kimura A, Ohta T. Inhibition of Inhibitor of Nuclear Factor-κB Phosphorylation Increases the Efficacy of Paclitaxel in in Vitro and in Vivo Ovarian Cancer Models. Clin Cancer Res. Available from: https://aacrjournals.org/clincancerres/article/10/22/7645/185396/Inhibition-of-Inhibitor-of-Nuclear-Factor-B 10.1158/1078-0432.CCR-04-0958 15569997

[70] Martincuks A, Song J, Kohut A, Zhang C, Li Y-J, Zhao Q. PARP inhibition activates STAT3 in both tumor and immune cells underlying therapy resistance and immunosuppression in ovarian cancer. Front Oncol. 11. 10.3389/fonc.2021.724104 PMC8693573 34956861

[71] Stronach EA, Alfraidi A, Rama N, Datler C, Studd JB, Agarwal R. HDAC4-Regulated STAT1 activation mediates platinum resistance in ovarian cancer. Cancer Res 71:4412–22. 10.1158/0008-5472.CAN-10-4111 PMC3130134 21571862

[72] Wang Z, Chen W, Zuo L, Xu M, Wu Y, Huang J. The Fibrillin-1/VEGFR2/STAT2 signaling axis promotes chemoresistance via modulating Glycolysis and angiogenesis in ovarian cancer organoids and cells. Cancer Commun 42:245–65. 10.1002/cac2.12274 PMC8923131 35234370

[73] Chen M-W, Yang S-T, Chien M-H, Hua K-T, Wu C-J, Hsiao SM. The STAT3-miRNA-92-Wnt signaling pathway regulates spheroid formation and malignant progression in ovarian cancer. Cancer Res 77:1955–67. 10.1158/0008-5472.CAN-16-1115 28209618

[74] Ji T, Gong D, Han Z, Wei X, Yan Y, Ye F. Abrogation of constitutive Stat3 activity circumvents cisplatin resistant ovarian cancer. Cancer Lett 341:231–9. 10.1016/j.canlet.2013.08.022 23962558

[75] Duan Z, Foster R, Bell DA, Mahoney J, Wolak K, Vaidya A. Signal transducers and activators of transcription 3 pathway activation in Drug-Resistant ovarian cancer. Clin Cancer Res 12:5055–63. 10.1158/1078-0432.CCR-06-0861 16951221

[76] Yang P-L, Liu L-X, Li E-M, Xu L-Y. STAT3, the Challenge for Chemotherapeutic and Radiotherapeutic Efficacy. 10.3390/cancers12092459 PMC7564975 32872659

[77] Lu W, Chen H, Yel F, Wang F, Xie X. VEGF induces phosphorylation of STAT3 through binding VEGFR2 in ovarian carcinoma cells in vitro. Eur J Gynaecol Oncol 27:363–9. 17009627

[78] Yan H, Guo B-Y, Zhang S. Cancer-associated fibroblasts attenuate Cisplatin-induced apoptosis in ovarian cancer cells by promoting STAT3 signaling. Biochem Biophys Res Commun 470:947–54. 10.1016/j.bbrc.2016.01.131 26826383

[79] Han Z, Feng J, Hong Z, Chen L, Li W, Liao S. Silencing of the STAT3 signaling pathway reverses the inherent and induced chemoresistance of human ovarian cancer cells. Biochem Biophys Res Commun 435:188–94. 10.1016/j.bbrc.2013.04.087 23665025

[80] Giordano M, Decio A, Battistini C, Baronio M, Bianchi F, Villa A. L1CAM promotes ovarian cancer stemness and tumor initiation via FGFR1/SRC/STAT3 signaling. J Exp Clin Cancer Res. Available from: https://jeccr.biomedcentral.com/articles/10.1186/s13046-021-02117-z 10.1186/s13046-021-02117-z PMC8513260 34645505

[81] Yin S, Yang L, Zheng Y, Zang R. Wip1 suppresses angiogenesis through the STAT3-VEGF signalling pathway in serous ovarian cancer. J Ovarian Res. 15. Available from: https://ovarianresearch.biomedcentral.com/articles/10.1186/s13048-022-00990-6 10.1186/s13048-022-00990-6 PMC9087943 35538489

[82] Martincuks A, Song J, Kohut A, Zhang C, Li Y-J, Zhao Q. PARP Inhibition Activates STAT3 in Both Tumor and Immune Cells Underlying Therapy Resistance and Immunosuppression In Ovarian Cancer. Front Oncol. Available from: https://www.frontiersin.org/articles/10.3389/fonc.2021.724104/full 10.3389/fonc.2021.724104 PMC8693573 34956861

[83] Jin P, Liu Y, Wang R. STAT3 regulated miR-216a promotes ovarian cancer proliferation and cisplatin resistance. Biosci Rep. Available from: https://portlandpress.com/bioscirep/article/38/4/BSR20180547/58183/STAT3-regulated-miR-216a-promotes-ovarian-cancer 10.1042/BSR20180547 PMC6131203 30061175

[84] Liang F, Ren C, Wang J, Wang S, Yang L, Han X. The crosstalk between STAT3 and p53/RAS signaling controls cancer cell metastasis and cisplatin resistance via the Slug/MAPK/PI3K/AKT-mediated regulation of EMT and autophagy. Oncogenesis. 8. 10.1038/s41389-019-0165-8 PMC6785561 31597912

[85] Wang L, Zhang F, Cui J, Chen L, Chen Y, Liu B. CAFs enhance Paclitaxel resistance by inducing EMT through the IL 6/JAK2/STAT3 pathway. Oncol Rep. 10.3892/or.2018.6311 PMC5928760 29565447

[86] Roberts CM, Tran MA, Pitruzzello MC, Wen W, Loeza J, Dellinger TH. TWIST1 drives cisplatin resistance and cell survival in an ovarian cancer model, via upregulation of GAS6, L1CAM, and Akt signalling. Sci Rep. 6. 10.1038/srep37652 PMC5120297 27876874

[87] Yue P, Zhang X, Paladino D, Sengupta B, Ahmad S, Holloway RW. Hyperactive EGF receptor, Jaks and Stat3 signaling promote enhanced colony-forming ability, motility and migration of cisplatin-resistant ovarian cancer cells. Oncogene. Available from: https://www.nature.com/articles/onc2011409 10.1038/onc.2011.409 PMC3245777 21909139

[88] Perez-Fidalgo JA, Ortega B, Simon S, Samartzis EP, Boussios S. NOTCH signalling in ovarian cancer angiogenesis. Ann Transl Med 8:1705–1705. 10.21037/atm-20-4497 PMC7812236 33490217

[89] Zhou Y, Chen Q, Qin R, Zhang K, Li H. MicroRNA-449a reduces cell survival and enhances cisplatin-induced cytotoxicity via downregulation of NOTCH1 in ovarian cancer cells. Tumor Biol 35:12369–78. 10.1007/s13277-014-2551-3 25179844

[90] ZOU W, MA X, HUA W. CHEN B, CAI G. Caveolin-1 mediates chemoresistance in cisplatin-resistant ovarian cancer cells by targeting apoptosis through the Notch-1/Akt/NF-κB pathway. Oncol Rep 34:3256–63. 10.3892/or.2015.4320 26503358

[91] WANG M, MA X, WANG J, WANG L, WANG Y. Pretreatment with the γ-secretase inhibitor DAPT sensitizes drug-resistant ovarian cancer cells to cisplatin by downregulation of Notch signaling. Int J Oncol. 44:1401–9. 10.3892/ijo.2014.2301 24535252

[92] Steg AD, Katre AA, Goodman B, Han H-D, Nick AM, Stone RL. Targeting the Notch Ligand Jagged1 in Both Tumor Cells and Stroma in Ovarian Cancer. Clin Cancer Res. Available from: https://aacrjournals.org/clincancerres/article/17/17/5674/76500/Targeting-the-Notch-Ligand-Jagged1-in-Both-Tumor 10.1158/1078-0432.CCR-11-0432 PMC3166981 21753153

[93] Alniaimi AN, Demorest-Hayes K, Alexander VM, Seo S, Yang D, Rose S. Increased Notch1 Expression Is Associated With PoorOverall Survival in Patients WithOvarian Cancer. Int J Gynecol Cancer. Available from: https://linkinghub.elsevier.com/retrieve/pii/S1048891X24015883 10.1097/IGC.0000000000000359 25611897

[94] Ceccarelli S, Megiorni F, Bellavia D, Marchese C, Screpanti I, Checquolo S. Notch3 targeting: A novel weapon against ovarian cancer stem cells. Stem Cells Int. 2019:1–8. 10.1155/2019/6264931 PMC6339748 30723507

[95] Kim MJ, Kim A-R, Jeong J-Y, Kim K-I, Kim T-H, Lee C. Correlation of ALDH1 and Notch3 expression: clinical implication in ovarian carcinomas. J Cancer. 8:3331–42. 10.7150/jca.18955 PMC5665050 29158806

[96] Xu Y, Miao C, Jin C, Qiu C, Li Y, Sun X. SUSD2 promotes cancer metastasis and confers cisplatin resistance in high grade serous ovarian cancer. Exp Cell Res. 10.1016/j.yexcr.2017.12.029 29305171

[97] Rahman MT, Nakayama K, Rahman M, Katagiri H, Katagiri A, Ishibashi T. Notch3 Overexpression as Potential Therapeutic Target in Advanced Stage Chemoresistant Ovarian Cancer. Am J Clin Pathol. Available from: https://academic.oup.com/ajcp/article/138/4/535/1760794 10.1309/AJCPKDLRQ8F3EWNS 23010708

[98] Hu W, Lu C, Dong HH, Huang J, Shen D, Stone RL. Biological Roles of the Delta Family Notch Ligand Dll4 in Tumor and Endothelial Cells in Ovarian Cancer. Cancer Res. Available from: https://aacrjournals.org/cancerres/article/71/18/6030/568030/Biological-Roles-of-the-Delta-Family-Notch-Ligand 10.1158/0008-5472.CAN-10-2719 PMC3174342 21795478

[99] Fu S, Corr BR, Culm-Merdek K, Mockbee C, Youssoufian H, Stagg R. Phase Ib study of navicixizumab plus Paclitaxel in patients with Platinum-Resistant ovarian, primary peritoneal, or fallopian tube cancer. J Clin Oncol 40:2568–77. 10.1200/JCO.21.01801 PMC9362870 35439029

[100] Coleman RL, Handley KF, Burger R, Molin GZD, Stagg R, Sood AK. Demcizumab combined with Paclitaxel for platinum-resistant ovarian, primary peritoneal, and fallopian tube cancer: the SIERRA open-label phase Ib trial. Gynecol Oncol. 157:386–91. 10.1016/j.ygyno.2020.01.042 32037195

[101] Principe DR, Doll JA, Bauer J, Jung B, Munshi HG, Bartholin L et al. TGF-: Duality of Function Between Tumor Prevention and Carcinogenesis. JNCI J Natl Cancer Inst. 2014;106:djt369–djt369. Available from: https://academic.oup.com/jnci/article-lookup/doi/10.1093/jnci/djt369 10.1093/jnci/djt369 PMC3952197 24511106

[102] Landen CN, Birrer MJ, Sood AK. Early events in the pathogenesis of epithelial ovarian cancer. J Clin Oncol. 26:995–1005. 10.1200/JCO.2006.07.9970 18195328

[103] Sunde JS, Donninger H, Wu K, Johnson ME, Pestell RG, Rose GS. Expression profiling identifies altered expression of genes that contribute to the Inhibition of transforming growth Factor-β signaling in ovarian cancer. Cancer Res 66:8404–12. 10.1158/0008-5472.CAN-06-0683 16951150

[104] Helleman J, Jansen MPHM, Burger C, Burg MEL, Berns EMJJ. Integrated genomics of chemotherapy resistant ovarian cancer: A role for extracellular matrix, TGFbeta and regulating MicroRNAs. Int J Biochem Cell Biol 42:25–30. 10.1016/j.biocel.2009.10.016 19854294

[105] Wang C-W, Lee B-H, Tai C-J. The Inhibition of cordycepin on cancer stemness in TGF-beta induced chemo-resistant ovarian cancer cell. Oncotarget 8:111912–21. 10.18632/oncotarget.22951 PMC5762368 29340100

[106] Roane BM, Arend RC, Birrer MJ. Review: Targeting the Transforming Growth Factor-Beta Pathway in Ovarian Cancer. Available from: https://www.mdpi.com/2072-6694/11/5/668 10.3390/cancers11050668 PMC6562901 31091744

[107] Gao Y, Shan N, Zhao C, Wang Y, Xu F, Li J. LY2109761 enhances cisplatin antitumor activity in ovarian cancer cells. Int J Clin Exp Pathol. Available from: http://www.ncbi.nlm.nih.gov/pubmed/26191185 PMC4503057 26191185

[108] Mitra T, Prasad P, Mukherjee P, Chaudhuri SRC, U R. S.S. Stemness and chemoresistance are imparted to the OC cells through TGFβ1 driven EMT. J Cell Biochem. Available from: https://onlinelibrary.wiley.com/doi/10.1002/jcb.26753 10.1002/jcb.26753 29537103

[109] Wu X, Zhao J, Ruan Y, Sun L, Xu C, Jiang H. Sialyltransferase ST3GAL1 promotes cell migration, invasion, and TGF-β1-induced EMT and confers paclitaxel resistance in ovarian cancer. Cell Death Dis. Available from: https://www.nature.com/articles/s41419-018-1101-0 10.1038/s41419-018-1101-0 PMC6207573 30375371

[110] Wang Y, Xiang J, Wang J, Ji Y. Downregulation of TGF-β1 suppressed proliferation and increased chemosensitivity of ovarian cancer cells by promoting BRCA1/Smad3 signaling. Biol Res. 51. 10.1186/s40659-018-0205-4 PMC6310971 30594239

[111] Zhu H, Gu X, Xia L, Zhou Y, Bouamar H, Yang J. A novel TGFβ trap blocks Chemotherapeutics-Induced TGFβ1 signaling and enhances their anticancer activity in gynecologic cancers. Clin Cancer Res 24:2780–93. 10.1158/1078-0432.CCR-17-3112 PMC6004245 29549162

[112] González Muñoz T, Amaral AT, Puerto-Camacho P, Peinado H, de Álava E. Endoglin in the spotlight to treat cancer. Int J Mol Sci. 2021;22:3186. 33804796 10.3390/ijms22063186 PMC8003971

[113] Dallas NA, Samuel S, Xia L, Fan F, Gray MJ, Lim SJ. Endoglin (CD105): A marker of tumor vasculature and potential target for therapy. Clin Cancer Res 14:1931–7. 10.1158/1078-0432.CCR-07-4478 18381930

[114] Zhang J, Yuan B, Zhang H, Li H. Human epithelial ovarian cancer cells expressing CD105, CD44 and CD106 surface markers exhibit increased invasive capacity and drug resistance. Oncol Lett. 10.3892/ol.2019.10221 PMC6507388 31186752

[115] Bai S, Zhu W, Coffman L, Vlad A, Schwartz LE, Elishaev E. CD105 is expressed in ovarian cancer precursor lesions and Is Required for Metastasis to the Ovary. 10.3390/cancers11111710 PMC6896092 31684072

[116] Garcia AA, Makker V, Spitz DL, Matei DE, Nick AM, Landen CN. Trc105 (Anti-Endoglin Antibody) in Combination with Bevacizumab (Bev) and As a Single Agent for Platinum Resistant Ovarian Cancer. Ann Oncol.

[117] Colic E, Patel PU, Kent OA, Aberrant MAPK. Signaling Offers Therapeutic Potential for Treatment of Ovarian Carcinoma. Onco Targets Ther. 2022;Volume 15:1331–46. Available from: https://www.dovepress.com/aberrant-mapk-signaling-offers-therapeutic-potential-for-treatment-of--peer-reviewed-fulltext-article-OTT 10.2147/OTT.S361512 PMC9645123 36388156

[118] Emmanuel C, Chiew Y-E, George J, Etemadmoghadam D, Anglesio MS, Sharma R. Genomic Classification of Serous Ovarian Cancer with Adjacent Borderline Differentiates RAS Pathway and TP53 -Mutant Tumors and Identifies NRAS as an Oncogenic Driver. Clin Cancer Res. Available from: https://aacrjournals.org/clincancerres/article/20/24/6618/121290/Genomic-Classification-of-Serous-Ovarian-Cancer 10.1158/1078-0432.CCR-14-1292 25316818

[119] Banerjee S, Kaye SB. New Strategies in the Treatment of Ovarian Cancer: Current Clinical Perspectives and Future Potential. Clin Cancer Res. Available from: https://aacrjournals.org/clincancerres/article/19/5/961/78457/New-Strategies-in-the-Treatment-of-Ovarian-Cancer 10.1158/1078-0432.CCR-12-2243 23307860

[120] Manning-Geist B, Gordhandas S, Liu YL, Zhou Q, Iasonos A, Cruz Paula A. MAPK pathway genetic alterations are associated with prolonged overall survival in Low-Grade serous ovarian carcinoma. Clin Cancer Res 28:4456–65. 10.1158/1078-0432.CCR-21-4183 PMC9582036 35443055

[121] Lee S, Yoon S, Kim D-H. A high nuclear basal level of ERK2 phosphorylation contributes to the resistance of cisplatin-resistant human ovarian cancer cells. Gynecol Oncol. 104:338–44. 10.1016/j.ygyno.2006.08.040 17023032

[122] Ghoneum A, Almousa S, Warren B, Abdulfattah AY, Shu J, Abouelfadl H. Exploring the clinical value of tumor microenvironment in platinum-resistant ovarian cancer. Semin Cancer Biol 77:83–98. 10.1016/j.semcancer.2020.12.024 PMC8286277 33476723

[123] Hayakawa J, Ohmichi M, Kurachi H, Kanda Y, Hisamoto K, Nishio Y. Inhibition of BAD phosphorylation either at Serine 112 via extracellular signal-regulated protein kinase cascade or at Serine 136 via Akt cascade sensitizes human ovarian cancer cells to cisplatin. Cancer Res 60:5988–94. 11085518

[124] Cui W, Yazlovitskaya EM, Mayo MS, Pelling JC, Persons DL. Cisplatin-induced response of c-jun N-terminal kinase 1 and extracellular signal–regulated protein kinases 1 and 2 in a series of cisplatin-resistant ovarian carcinoma cell lines. Mol Carcinog 29:219–28. 11170260

[125] Bhatt M, Ivan C, Xie X, Siddik ZH. Drug-dependent functionalization of wild-type and mutant p53 in cisplatin-resistant human ovarian tumor cells. Oncotarget. Available from: https://www.oncotarget.com/lookup/doi/10.18632/oncotarget.14228 10.18632/oncotarget.14228 PMC5355233 28038466

[126] Gebauer G, Mirakhur B, Nguyen Q, Shore SK, Simpkins H, Dhanasekaran N. Cisplatin-resistance involves the defective processing of MEKK1 in human ovarian adenocarcinoma 2008/C13 cells. Int J Oncol. Available from: http://www.spandidos-publications.com/10.3892/ijo.16.2.321 10.3892/ijo.16.2.321 10639576

[127] Sun C, Fang Y, Yin J, Chen J, Ju Z, Zhang D. Rational combination therapy with PARP and MEK inhibitors capitalizes on therapeutic liabilities in RAS mutant cancers. Sci Transl Med. Available from: https://www.science.org/doi/10.1126/scitranslmed.aal5148 10.1126/scitranslmed.aal5148 PMC5919217 28566428

[128] Vena F, Jia R, Esfandiari A, Garcia-Gomez JJ, Rodriguez-Justo M, Ma J. MEK inhibition leads to BRCA2 downregulation and sensitization to DNA damaging agents in pancreas and ovarian cancer models. Oncotarget. Available from: https://www.oncotarget.com/lookup/doi/10.18632/oncotarget.24294 10.18632/oncotarget.24294 PMC5837749 29545922

[129] Bussies PL, Schlumbrecht M. Dual Fulvestrant-Trametinib Therapy in Recurrent Low-Grade Serous Ovarian Cancer. Oncol. Available from: https://academic.oup.com/oncolo/article/25/7/e1124/6443619 10.1634/theoncologist.2020-0101 PMC7356701 32369640

[130] Grisham RN, Moore KN, Gordon MS, Harb W, Cody G, Halpenny DF. Phase Ib Study of Binimetinib with Paclitaxel in Patients with Platinum-Resistant Ovarian Cancer: Final Results, Potential Biomarkers, and Extreme Responders. Clin Cancer Res. Available from: https://aacrjournals.org/clincancerres/article/24/22/5525/81052/Phase-Ib-Study-of-Binimetinib-with-Paclitaxel-in 10.1158/1078-0432.CCR-18-0494 PMC6616528 29844129

[131] Wang J, Kho DH, Zhou J-Y, Davis RJ, Wu GS. MKP-1 suppresses PARP-1 degradation to mediate cisplatin resistance. Oncogene 36:5939–47. 10.1038/onc.2017.197 PMC5658239 28650468

[132] Wang J, Zhou J-Y, Wu GS. ERK-Dependent MKP-1–Mediated cisplatin resistance in human ovarian cancer cells. Cancer Res 67:11933–41. 10.1158/0008-5472.CAN-07-5185 18089824

[133] Liu S, Zou B, Tian T, Luo X, Mao B, Zhang X. Overexpression of the LncRNA FER1L4 inhibits Paclitaxel tolerance of ovarian cancer cells via the regulation of the MAPK signaling pathway. J Cell Biochem 120:7581–9. 10.1002/jcb.28032 30444026

[134] Arend RC, Londoño-Joshi AI, Straughn JM, Buchsbaum DJ. The Wnt/β-catenin pathway in ovarian cancer: A review. Gynecol Oncol. 131:772–9. 10.1016/j.ygyno.2013.09.034 24125749

[135] Cannon M, Ghosh D, Gujja S. Signaling circuits and regulation of immune suppression by ovarian Tumor-Associated macrophages. Vaccines. 10.3390/vaccines3020448 PMC4494355 26343197

[136] Zhang S, Balch C, Chan MW, Lai H-C, Matei D, Schilder JM. Identification and characterization of ovarian Cancer-Initiating cells from primary human tumors. Cancer Res. 10.1158/0008-5472.CAN-08-0364 PMC2553722 18519691

[137] Schindler AJ, Watanabe A, Howell SB. LGR5 and LGR6 in stem cell biology and ovarian cancer. Oncotarget 9:1346–55. 10.18632/oncotarget.20178 PMC5787443 29416699

[138] de Lau W, Peng WC, Gros P, Clevers H. The R-spondin/Lgr5/Rnf43 module: regulator of Wnt signal strength. Genes Dev. 2014;28:305–16. 24532711 10.1101/gad.235473.113 PMC3937510

[139] Liu Q, Zhao Y, Xing H, Li L, Li R, Dai J. The role of R-spondin 1 through activating Wnt/β-catenin in the growth, survival and migration of ovarian cancer cells. Gene. 10.1016/j.gene.2018.11.098 30572097

[140] Yamamoto TM, McMellen A, Watson ZL, Aguilera J, Ferguson R, Nurmemmedov E. Activation of Wnt signaling promotes Olaparib resistant ovarian cancer. Mol Carcinog 58:1770–82. 10.1002/mc.23064 PMC7537108 31219654

[141] Zhang H, Qiu J, Ye C, Yang D, Gao L, Su Y. ROR1 expression correlated with poor clinical outcome in human ovarian cancer. Sci Rep. 4. 10.1038/srep05811 PMC4108928 25056203

[142] Zhang S, Cui B, Lai H, Liu G, Ghia EM, Widhopf GF. Ovarian cancer stem cells express ROR1, which can be targeted for anti–cancer-stem-cell therapy. Proc Natl Acad Sci. 10.1073/pnas.1419599111 PMC4260559 25411317

[143] Moore KN, Gunderson CC, Sabbatini P, McMeekin DS, Mantia-Smaldone G, Burger RA. A phase 1b dose escalation study of Ipafricept (OMP 54F28) in combination with Paclitaxel and carboplatin in patients with recurrent platinum-sensitive ovarian cancer. Gynecol Oncol. 154:294–301. 10.1016/j.ygyno.2019.04.001 PMC7397408 31174889

[144] Kanlikilicer P, Ozpolat B, Aslan B, Bayraktar R, Gurbuz N, Rodriguez-Aguayo C. Therapeutic targeting of AXL receptor tyrosine kinase inhibits tumor growth and intraperitoneal metastasis in ovarian cancer models. Mol Ther Nucleic Acids 9:251–62. 10.1016/j.omtn.2017.06.023 PMC5675720 29246304

[145] Rea K, Pinciroli P, Sensi M, Alciato F, Bisaro B, Lozneanu L. Novel Axl-driven signaling pathway and molecular signature characterize high-grade ovarian cancer patients with poor clinical outcome. Oncotarget 6:30859–75. 10.18632/oncotarget.5087 PMC4741573 26356564

[146] Buehler M, Tse B, Leboucq A, Jacob F, Caduff R, Fink D. Meta-Analysis of microarray data identifies GAS6 expression as an independent predictor of poor survival in ovarian cancer. Biomed Res Int. 2013:1–9. 10.1155/2013/238284 PMC3710590 23878800

[147] Mullen MM, Lomonosova E, Toboni MD, Oplt A, Cybulla E, Blachut B. GAS6/AXL Inhibition enhances ovarian cancer sensitivity to chemotherapy and PARP Inhibition through increased DNA damage and enhanced replication stress. Mol Cancer Res 20:265–79. 10.1158/1541-7786.MCR-21-0302 PMC8816812 34670865

[148] Linger RMA, Keating AK, Earp HS, Graham DK. TAM Receptor Tyrosine Kinases: Biologic Functions, Signaling, and Potential Therapeutic Targeting in Human Cancer. pp. 35–83. 10.1016/S0065-230X(08)00002-X PMC3133732 18620092

[149] Byers LA, Diao L, Wang J, Saintigny P, Girard L, Peyton M. An Epithelial–Mesenchymal transition gene signature predicts resistance to EGFR and PI3K inhibitors and identifies Axl as a therapeutic target for overcoming EGFR inhibitor resistance. Clin Cancer Res. 10.1158/1078-0432.CCR-12-1558 PMC3567921 23091115

[150] Antony J, Tan TZ, Kelly Z, Low J, Choolani M, Recchi C. The GAS6-AXL signaling network is a mesenchymal (Mes) molecular subtype–specific therapeutic target for ovarian cancer. Sci Signal. 9. 10.1126/scisignal.aaf8175 27703030

[151] Kariolis MS, Miao YR, Jones DS, Kapur S, Mathews IIG. A.J. An engineered Axl decoy receptor effectively silences the Gas6-Axl signaling axis. Nat Chem Biol 10:977–83. 10.1038/nchembio.1636 PMC4372605 25242553

[152] Yeo XH, Sundararajan V, Wu Z, Phua ZJC, Ho YY, Peh KLE. The effect of Inhibition of receptor tyrosine kinase AXL on DNA damage response in ovarian cancer. Commun Biol. 6. 10.1038/s42003-023-05045-0 PMC10287694 37349576

[153] Tanaka M, Siemann DW. Gas6/Axl signaling pathway in the tumor immune microenvironment. Cancers (Basel). 10.3390/cancers12071850 PMC7408754 32660000

[154] Quinn JM, Greenwade MM, Palisoul ML, Opara G, Massad K, Guo L. Therapeutic Inhibition of the receptor tyrosine kinase AXL improves sensitivity to platinum and taxane in ovarian cancer. Mol Cancer Ther 18:389–98. 10.1158/1535-7163.MCT-18-0537 PMC6363844 30478151

[155] Macleod K, Mullen P, Sewell J, Rabiasz G, Lawrie S, Miller E. Altered erbb receptor signaling and gene expression in Cisplatin-Resistant ovarian cancer. Cancer Res. 10.1158/0008-5472.CAN-04-2684 16061661

[156] Mullen MM, Lomonosova E, Toboni MD, Noia H, Wilke D, Oplt A. Inducing BRCAness by inhibiting the GAS6/AXL pathway in high-grade serous ovarian cancer. Gynecol Oncol 159:137–8.

[157] Tian M, Chen X, Li L, Wu H, Zeng D, Wang X. Inhibition of AXL enhances chemosensitivity of human ovarian cancer cells to cisplatin via decreasing Glycolysis. Acta Pharmacol Sin. 42:1180–9. 10.1038/s41401-020-00546-8 PMC8209001 33149145

[158] Knisely A, Hinchcliff EM, Gardiner E, Rangwala R, Lito K, Fellman B. Phase 1b study of Batiraxcept in combination with durvalumab in patients with platinum-resistant ovarian cancer. 10.1016/j.isci.2024.109801 PMC11079458 38726365

[159] Rocconi R. Hedgehog signaling pathway regulates the growth of ovarian cancer spheroid forming cells. Int J Oncol. 10.3892/ijo.2011.1093 21701772

[160] Chen X, Horiuchi A, Kikuchi N, Osada R, Yoshida J, Shiozawa T. Hedgehog signal pathway is activated in ovarian carcinomas, correlating with cell proliferation: it’s Inhibition leads to growth suppression and apoptosis. Cancer Sci 98:68–76. 10.1111/j.1349-7006.2006.00353.x PMC11158570 17083567

[161] Liao X, Siu MKY, Au CWH, Wong ESY, Chan HY, Ip PPC. Aberrant activation of Hedgehog signaling pathway in ovarian cancers: effect on prognosis, cell invasion and differentiation. Carcinogenesis. 10.1093/carcin/bgn230 PMC7109814 19028702

[162] Song X, Yan L, Lu C, Zhang C, Zhu F, Yang J. Activation of Hedgehog signaling and its association with cisplatin resistance in ovarian epithelial tumors. Oncol Lett. 10.3892/ol.2018.8008 PMC5840551 29552194

[163] Kudo K, Gavin E, Das S, Amable L, Shevde LA, Reed E. Inhibition of Gli1 results in altered c-Jun activation, Inhibition of cisplatin-induced upregulation of ERCC1, XPD and XRCC1, and Inhibition of platinum–DNA adduct repair. Oncogene 31:4718–24. 10.1038/onc.2011.610 22266871

[164] AMABLE L, GAVIN E, KUDO K, MENG E, ROCCONI RP. SHEVDE LA. GLI1 upregulates C-JUN through a specific 130-kDa isoform. Int J Oncol 44:655–61. 10.3892/ijo.2013.2222 PMC3928471 24366538

[165] Steg AD, Katre AA, Bevis KS, Ziebarth A, Dobbin ZC, Shah MM. Smoothened antagonists reverse taxane resistance in ovarian cancer. Mol Cancer Ther 11:1587–97. 10.1158/1535-7163.MCT-11-1058 PMC3392529 22553355

[166] Stathis A, Hess D, Moos R, Homicsko K, Griguolo G, Joerger M. Phase I trial of the oral smoothened inhibitor sonidegib in combination with Paclitaxel in patients with advanced solid tumors. Invest New Drugs 35:766–72. 10.1007/s10637-017-0454-z 28317088

[167] Zhang H, Hu L, Cheng M, Wang Q, Hu X, Chen Q. The Hedgehog signaling pathway promotes chemotherapy resistance via multidrug resistance protein 1 in ovarian cancer. Oncol Rep. 10.3892/or.2020.7798 PMC7640363 33125122

[168] Chen Y, Bieber MM, Teng NNH. Hedgehog signaling regulates drug sensitivity by targeting ABC transporters ABCB1 and ABCG2 in epithelial ovarian cancer. Mol Carcinog. 10.1002/mc.22015 23423781

[169] Hwang H-W, Mendell JT. MicroRNAs in cell proliferation, cell death, and tumorigenesis. Br J Cancer. 2006;94:776–80. Available from: https://www.nature.com/articles/6603023 10.1038/sj.bjc.6603023 PMC2361377 16495913

[170] Putri HMA, Novianti P, Pradjatmo H, Haryana S. MicroRNA–mediated approaches in ovarian cancer therapy: A comprehensive systematic review. Oncol Lett. 2024;28:491. Available from: http://www.spandidos-publications.com/10.3892/ol.2024.14624 10.3892/ol.2024.14624 PMC11342411 39185494

[171] Ha T-Y. MicroRNAs in human diseases: from cancer to cardiovascular disease. Immune Netw 11:135–54. 10.4110/in.2011.11.3.135 PMC3153666 21860607

[172] Peng Y, Croce CM. The role of MicroRNAs in human cancer. Signal Transduct Target Ther. 1. 10.1038/sigtrans.2015.4 PMC5661652 29263891

[173] Zhao L, Liang X, Wang L, Zhang X. The role of MiRNA in ovarian cancer: an overview. Reprod Sci 29:2760–7. 10.1007/s43032-021-00717-w PMC9537199 34973152

[174] Nguyen VHL, Yue C, Du KY, Salem M, O’Brien J, Peng C. The Role of microRNAs in Epithelial Ovarian Cancer Metastasis. Int J Mol Sci. 2020;21:7093. Available from: https://www.mdpi.com/1422-0067/21/19/7093 10.3390/ijms21197093 PMC7583982 32993038

[175] Saburi A, Kahrizi MS, Naghsh N, Etemadi H, İlhan A, Adili A. A comprehensive survey into the role of microRNAs in ovarian cancer chemoresistance; an updated overview. J Ovarian Res. Available from: https://ovarianresearch.biomedcentral.com/articles/10.1186/s13048-022-01012-1 10.1186/s13048-022-01012-1 PMC9264529 35799305

[176] Yang H, Kong W, He L, Zhao J-J, O’Donnell JD, Wang J. MicroRNA Expression Profiling in Human Ovarian Cancer: miR-214 Induces Cell Survival and Cisplatin Resistance by Targeting PTEN. Cancer Res. Available from: https://aacrjournals.org/cancerres/article/68/2/425/541232/MicroRNA-Expression-Profiling-in-Human-Ovarian 10.1158/0008-5472.CAN-07-2488 18199536

[177] Bendoraite A, Knouf EC, Garg KS, Parkin RK, Kroh EM, O’Briant KC et al. Regulation of miR-200 family microRNAs and ZEB transcription factors in ovarian cancer: Evidence supporting a mesothelial-to-epithelial transition. Gynecol Oncol. 2010;116:117–25. Available from: https://linkinghub.elsevier.com/retrieve/pii/S0090825809005976 10.1016/j.ygyno.2009.08.009 PMC2867670 19854497

[178] Asl ER, Sarabandi S, Shademan B, Dalvandi K, Sheikhansari G, Nourazarian A. MicroRNA targeting: A novel therapeutic intervention for ovarian cancer. Biochem Biophys Reports. 2023;35:101519. Available from: https://linkinghub.elsevier.com/retrieve/pii/S2405580823001000 10.1016/j.bbrep.2023.101519 PMC10382632 37521375

[179] Bhardwaj V, Sharma A, Parambath SV, Gul I, Zhang X, Lobie PE et al. Machine Learning for Endometrial Cancer Prediction and Prognostication. Front Oncol. 2022;12. Available from: https://www.frontiersin.org/articles/10.3389/fonc.2022.852746/full 10.3389/fonc.2022.852746 PMC9365068 35965548

[180] Stronach EA, Chen M, Maginn EN, Agarwal R, Mills GB, Wasan H. DNA-PK Mediates AKT Activation and Apoptosis Inhibition in Clinically Acquired Platinum Resistance. Neoplasia. Available from: https://linkinghub.elsevier.com/retrieve/pii/S147655861180093X 10.1593/neo.111032 PMC3223610 22131882

[181] Yang X, Fraser M, Abedini MR, Bai T, Tsang BK. Regulation of apoptosis-inducing factor-mediated, cisplatin-induced apoptosis by Akt. Br J Cancer. 98:803–8. 10.1038/sj.bjc.6604223 PMC2259169 18283299

[182] Choi HJ, Heo JH, Park JY, Jeong JY, Cho HJ, Park KS. A novel PI3K/mTOR dual inhibitor, CMG002, overcomes the chemoresistance in ovarian cancer. Gynecol Oncol. Available from: https://linkinghub.elsevier.com/retrieve/pii/S0090825819300459 10.1016/j.ygyno.2019.01.012 30686552

[183] Goto T, Takano M, Hirata J, Tsuda H. The involvement of FOXO1 in cytotoxic stress and drug-resistance induced by Paclitaxel in ovarian cancers. Br J Cancer. 98:1068–75. 10.1038/sj.bjc.6604279 PMC2275483 18319717

[184] Zhang M, Wang J, Guo Y, Yue H, Zhang L. Activation of PI3K/AKT/mTOR signaling axis by UBE2S inhibits autophagy leading to cisplatin resistance in ovarian cancer. J Ovarian Res. Available from: 10.1186/s13048-023-01314-y 10.1186/s13048-023-01314-y PMC10729389 38115063

[185] Xiao L, Peng Z, Zhu A, Xue R, Lu R, Mi J. Inhibition of RUNX1 promotes cisplatin-induced apoptosis in ovarian cancer cells. Biochem Pharmacol 180. 10.1016/j.bcp.2020.114116 32579960

[186] Momeny M, Yousefi H, Eyvani H, Moghaddaskho F, Salehi A, Esmaeili F. Blockade of nuclear factor-κB (NF-κB) pathway inhibits growth and induces apoptosis in chemoresistant ovarian carcinoma cells. Int J Biochem Cell Biol. Available from: https://linkinghub.elsevier.com/retrieve/pii/S1357272518300670 10.1016/j.biocel.2018.03.015 29567488

[187] Yan H, Guo B-Y, Zhang S. Cancer-associated fibroblasts attenuate Cisplatin-induced apoptosis in ovarian cancer cells by promoting STAT3 signaling. Biochem Biophys Res Commun. Available from: https://linkinghub.elsevier.com/retrieve/pii/S0006291X16301322 10.1016/j.bbrc.2016.01.131 26826383

[188] Ziebarth AJ, Nowsheen S, Steg AD, Shah MM, Katre AA, Dobbin ZC. Endoglin (CD105) contributes to platinum resistance and is A target for Tumor-Specific therapy in epithelial ovarian cancer. Clin Cancer Res 19:170–82. 10.1158/1078-0432.CCR-12-1045 PMC3537868 23147994

[189] Nodin B, Fridberg M, Uhlén M, Jirström K. Discovery of dachshund 2 protein as a novel biomarker of poor prognosis in epithelial ovarian cancer. J Ovarian Res. 5. 10.1186/1757-2215-5-6 PMC3295641 22284433

[190] Wang Y, Yang B, Zhao J, Yu X, Liu X, Zhang L. Epithelial mesenchymal transition induced by bone morphogenetic protein 9 hinders cisplatin efficacy in ovarian cancer cells. Available from: http://www.spandidos-publications.com/10.3892/mmr.2019.9814 10.3892/mmr.2019.9814 PMC6390058 30628686

[191] Ma H, Li Y, Wang X, Wu H, Qi G, Li R. PBK, targeted by EVI1, promotes metastasis and confers cisplatin resistance through inducing autophagy in high-grade serous ovarian carcinoma. Cell Death Dis. 10. 10.1038/s41419-019-1415-6 PMC6379381 30778048

[192] Wang Z, Xu J, Zhou J-Y, Liu Y, Wu GS. Mitogen-Activated Protein Kinase Phosphatase-1 Is Required for Cisplatin Resistance. Cancer Res. Available from: https://aacrjournals.org/cancerres/article/66/17/8870/526199/Mitogen-Activated-Protein-Kinase-Phosphatase-1-Is 10.1158/0008-5472.CAN-06-1280 16951204

[193] Chen M, Su J, Feng C, Liu Y, Zhao L, Tian Y. Chemokine CCL20 promotes the paclitaxel resistance of CD44 + CD117 + cells via the Notch1 signaling pathway in ovarian cancer. Available from: http://www.spandidos-publications.com/10.3892/mmr.2021.12274 10.3892/mmr.2021.12274 PMC8280726 34278466

[194] Yang J, Xing H, Lu D, Wang J, Li B, Tang J. Role of Jagged1/ STAT 3 signalling in platinum-resistant ovarian cancer. J Cell Mol Med. Available from: https://onlinelibrary.wiley.com/doi/10.1111/jcmm.14286 10.1111/jcmm.14286 PMC6533470 30993885

[195] McAuliffe SM, Morgan SL, Wyant GA, Tran LT, Muto KW, Chen YS. Targeting Notch, a key pathway for ovarian cancer stem cells, sensitizes tumors to platinum therapy. Proc Natl Acad Sci. Available from: 10.1073/pnas.1206400109 10.1073/pnas.1206400109 PMC3491453 23019585

[196] Gupta N, Xu Z, El-Sehemy A, Steed H, Fu Y. Notch3 induces epithelial–mesenchymal transition and attenuates carboplatin-induced apoptosis in ovarian cancer cells. Gynecol Oncol. 130:200–6. Available from: https://linkinghub.elsevier.com/retrieve/pii/S0090825813001741 10.1016/j.ygyno.2013.03.019 23542683

[197] Li H, Zhang W, Niu C, Lin C, Wu X, Jian Y. Nuclear orphan receptor NR2F6 confers cisplatin resistance in epithelial ovarian cancer cells by activating the Notch3 signaling pathway. Int J Cancer. Available from: https://onlinelibrary.wiley.com/doi/10.1002/ijc.32293 10.1002/ijc.32293 PMC6767785 30895619

[198] Nagaraj AB, Joseph P, Kovalenko O, Singh S, Armstrong A, Redline R. Critical role of Wnt/β-catenin signaling in driving epithelial ovarian cancer platinum resistance. Oncotarget. Available from: https://www.oncotarget.com/lookup/doi/10.18632/oncotarget.4690 10.18632/oncotarget.4690 PMC4695147 26125441

[199] Pratheeshkumar P, Divya SP, Parvathareddy SK, Alhoshani NM, Al-Badawi IA, Tulbah A. FoxM1 and β-catenin predicts aggressiveness in Middle Eastern ovarian cancer and their co-targeting impairs the growth of ovarian cancer cells. Oncotarget. Available from: https://www.oncotarget.com/lookup/doi/10.18632/oncotarget.23338 10.18632/oncotarget.23338 PMC5790485 29423068

[200] Mariya T, Hirohashi Y, Torigoe T, Tabuchi Y, Asano T, Saijo H. Matrix metalloproteinase-10 regulates stemness of ovarian cancer stem-like cells by activation of canonical Wnt signaling and can be a target of chemotherapy-resistant ovarian cancer. Oncotarget. Available from: https://www.oncotarget.com/lookup/doi/10.18632/oncotarget.8645 10.18632/oncotarget.8645 PMC5042016 27072580

[201] Wang Q, Wei X, Hu L, Zhuang L, Zhang H, Chen Q. Hedgehog– Gli2 signaling promotes chemoresistance in ovarian cancer cells by regulating MDR1. Front Oncol. 11. 10.3389/fonc.2021.794959 PMC8763667 35059317

[202] Dey A, Dhar Dwivedi SK, Wang L, Hossen MN, Neizer-Ashun F, Bieniasz M. Targeting BMI1 mitigates chemoresistance in ovarian cancer. Genes Dis. Available from: https://linkinghub.elsevier.com/retrieve/pii/S2352304222000460 10.1016/j.gendis.2022.02.006 PMC9485277 36157476

[203] Zhang Z, Zhang L, Wang B, Wei R, Wang Y, Wan J. MiR-337–3p suppresses proliferation of epithelial ovarian cancer by targeting PIK3CA and PIK3CB. Cancer Lett 469:54–67. 10.1016/j.canlet.2019.10.021 31629932

[204] WANG Z, TING Z, LI Y, CHEN G, LU Y, HAO X. microRNA-199a is able to reverse cisplatin resistance in human ovarian cancer cells through the Inhibition of mammalian target of Rapamycin. Oncol Lett 6:789–94. 10.3892/ol.2013.1448 PMC3789061 24137412

[205] Cui Y, Wu F, Tian D, Wang T, Lu T, Huang X. miR-199a-3p enhances cisplatin sensitivity of ovarian cancer cells by targeting ITGB8. Oncol Rep. 10.3892/or.2018.6259 PMC5868401 29436681

[206] Zhang L, Yu J, Leng Y, Zhu R, Liu H, Wang X. MiR-181c sensitizes ovarian cancer cells to Paclitaxel by targeting GRP78 through the PI3K/Akt pathway. Cancer Gene Ther 29:770–83. 10.1038/s41417-021-00356-y 34145425

[207] Guo P, Xiong X, Zhang S, Peng D. miR-100 resensitizes resistant epithelial ovarian cancer to cisplatin. Oncol Rep 36:3552–8. 10.3892/or.2016.5140 27748936

[208] Wu D, Lu P, Mi X, Miao J. Downregulation of miR-503 contributes to the development of drug resistance in ovarian cancer by targeting PI3K p85. Arch Gynecol Obs 297:699–707. 10.1007/s00404-018-4649-0 29327155

[209] Xu S, Fu G-B, Tao Z, OuYang J, Kong F, Jiang B-H. MiR-497 decreases cisplatin resistance in ovarian cancer cells by targeting mTOR/P70S6K1. Oncotarget 6:26457–71. 10.18632/oncotarget.4762 PMC4694914 26238185

[210] Qin X, Sun L, Wang J. Restoration of microRNA-708 sensitizes ovarian cancer cells to cisplatin via IGF2BP1/Akt pathway. Cell Biol Int 41:1110–8. 10.1002/cbin.10819 28685895

[211] Chen Y, Wang L, Zhou J. Effects of microRNA-1271 on ovarian cancer via Inhibition of epithelial‐mesenchymal transition and cisplatin resistance. J Obstet Gynaecol Res 45:2243–54. 10.1111/jog.14079 31411791

[212] Chen R, Alvero AB, Silasi DA, Kelly MG, Fest S, Visintin I. Regulation of IKKβ by miR-199a affects NF-κB activity in ovarian cancer cells. Oncogene 27:4712–23. 10.1038/onc.2008.112 PMC3041589 18408758

[213] Shuang T, Wang M, Zhou Y, Shi C, Wang D. NF-κB1, c-Rel, and ELK1 inhibit miR-134 expression leading to Table 1 upregulation in paclitaxel-resistant human ovarian cancer. Oncotarget 8:24853–68. 10.18632/oncotarget.15267 PMC5421894 28206956

[214] Park G, Bin KD. MicroRNA-503-5p Inhibits the CD97-Mediated JAK2/STAT3 Pathway in Metastatic or Paclitaxel-Resistant Ovarian Cancer Cells. Neoplasia. 2019;21:206–15. Available from: https://linkinghub.elsevier.com/retrieve/pii/S1476558618305104 10.1016/j.neo.2018.12.005 PMC6355618 30622051

[215] Zhu X, Shen H, Yin X, Long L, Chen X, Feng F. IL-6R/STAT3/miR-204 feedback loop contributes to cisplatin resistance of epithelial ovarian cancer cells. Oncotarget 8:39154–66. 10.18632/oncotarget.16610 PMC5503602 28388577

[216] Tian X, Zuo X, Hou M, Li C, Teng Y. LncRNA-H19 regulates chemoresistance to carboplatin in epithelial ovarian cancer through microRNA-29b-3p and STAT3. J Cancer. 12:5712–22. 10.7150/jca.58979 PMC8408112 34475985

[217] Jeong J-Y, Kang H, Kim TH, Kim G, Heo J-H, Kwon A-Y. MicroRNA-136 inhibits cancer stem cell activity and enhances the anti-tumor effect of Paclitaxel against chemoresistant ovarian cancer cells by targeting Notch3. Cancer Lett 386:168–78. 10.1016/j.canlet.2016.11.017 27887917

[218] Liu MX, Siu MKY, Liu SS, Yam JWP, Ngan HYS, Chan DW. Epigenetic Silencing of microRNA-199b-5p is associated with acquired chemoresistance via activation of JAG1-Notch1 signaling in ovarian cancer. Oncotarget 5:944–58. 10.18632/oncotarget.1458 PMC4011596 24659709

[219] Kim TH, Jeong J-Y, Park J-Y, Kim S-W, Heo JH, Kang H. miR-150 enhances apoptotic and anti-tumor effects of Paclitaxel in Paclitaxel-resistant ovarian cancer cells by targeting Notch3. Oncotarget 8:72788–800. 10.18632/oncotarget.20348 PMC5641169 29069826

[220] Xia B, Zhao Z, Wu Y, Wang Y, Zhao Y, Wang J. Circular RNA circTNPO3 regulates Paclitaxel resistance of ovarian cancer cells by miR-1299/NEK2 signaling pathway. Mol Ther Nucleic Acids. 21:780–91. 10.1016/j.omtn.2020.06.002 PMC7419276 32791450

[221] Pei Y, Li K, Lou X, Wu Y, Dong X, Wang W. MiR 1299/NOTCH3/TUG1 feedback loop contributes to the malignant proliferation of ovarian cancer. Oncol Rep 44:438–48. 10.3892/or.2020.7623 PMC7336509 32468036

[222] Cai Y, An B, Yao D, Zhou H, Zhu J. MicroRNA miR-30a inhibits cisplatin resistance in ovarian cancer cells through autophagy. Bioengineered 12:10713–22. 10.1080/21655979.2021.2001989 PMC8810079 34747309

[223] Yoshida K, Yokoi A, Sugiyama M, Oda S, Kitami K, Tamauchi S. Expression of the chrXq27.3 MiRNA cluster in recurrent ovarian clear cell carcinoma and its impact on cisplatin resistance. Oncogene 40:1255–68. 10.1038/s41388-020-01595-3 PMC7892337 33420363

[224] Han T, Chen T, Chen L, Li K, Xiang D, Dou L. HLF promotes ovarian cancer progression and chemoresistance via regulating Hippo signaling pathway. Cell Death Dis 14. 10.1038/s41419-023-06076-5 PMC10502110 37709768

[225] van Jaarsveld MTM, van Kuijk PF, Boersma AWM, Helleman J, van IJcken WF, Mathijssen RHJ et al. miR-634 restores drug sensitivity in resistant ovarian cancer cells by targeting the Ras-MAPK pathway. Mol Cancer. 2015;14:196. Available from: http://molecular-cancer.biomedcentral.com/articles/10.1186/s12943-015-0464-4 10.1186/s12943-015-0464-4 PMC4650519 26576679

[226] Wang J, Da C, Su Y, Song R, Bai Z. MKNK2 enhances chemoresistance of ovarian cancer by suppressing autophagy via miR-125b. Biochem Biophys Res Commun 556:31–8. 10.1016/j.bbrc.2021.02.084 33836345

[227] Jiang X, Cheng Y, He Y, Cong S, Sun L, Wu D. LNC00115 mediates cisplatin resistance by regulating the miR-7/ERK signalling pathway in ovarian cancer. Cancer Manag Res 13:3817–26. 10.2147/CMAR.S295097 PMC8123956 34007214

[228] Xu Z, Yao T, Liu W. miR-378a-3p sensitizes ovarian cancer cells to cisplatin through targeting MAPK1/GRB2. Biomed Pharmacother. 107:1410–7. 10.1016/j.biopha.2018.08.132 30257357

[229] Lin M, Xia B, Qin L, Chen H, Lou G. S100A7 regulates ovarian cancer cell metastasis and chemoresistance through MAPK signaling and is targeted by miR-330-5p. DNA Cell Biol. 10.1089/dna.2017.3953 29485916

[230] Hisamatsu T, McGuire M, Wu SY, Rupaimoole R, Pradeep S, Bayraktar E. PRKRA /PACT expression promotes chemoresistance of mucinous ovarian cancer. Mol Cancer Ther 18:162–72. 10.1158/1535-7163.MCT-17-1050 PMC6318044 30305341

[231] Ha C, Hu L, Ren Y, Yang J, Xin L. SND1 confers chemoresistance to cisplatin-induced apoptosis by targeting GAS6-AKT in SKOV3 ovarian cancer cells. Med Oncol. 39. 10.1007/s12032-022-01763-3 35972612

[232] Wang J, Hu Y, Ye C, Liu J. miR-1224-5p inhibits the proliferation and invasion of ovarian cancer via targeting SND1. Hum Cell. 2020;33:780–9. Available from: https://link.springer.com/10.1007/s13577-020-00364-4 10.1007/s13577-020-00364-4 32409958

[233] Zhu X, Shen H, Yin X, Long L, Xie C, Liu Y. miR-186 regulation of Twist1 and ovarian cancer sensitivity to cisplatin. Oncogene 35:323–32. 10.1038/onc.2015.84 25867064

[234] Niu Q, Liu Z, Gao J, Wang Q. MiR-338-3p enhances ovarian cancer cell sensitivity to cisplatin by downregulating WNT2B. Yonsei Med J. 60. 10.3349/ymj.2019.60.12.1146 PMC6881712 31769245

[235] Xiao S, Li Y, Pan Q, Ye M, He S, Tian Q. MiR-34c/SOX9 axis regulates the chemoresistance of ovarian cancer cell to cisplatin‐based chemotherapy. J Cell Biochem 120:2940–53. 10.1002/jcb.26865 30537410

[236] Ma J, Zhou C, Chen X. miR-636 inhibits EMT, cell proliferation and cell cycle of ovarian cancer by directly targeting transcription factor Gli2 involved in Hedgehog pathway. Cancer Cell Int. 21. 10.1186/s12935-020-01725-7 PMC7819188 33472614

[237] XIE Z, CAO L, ZHANG J. miR-21 modulates Paclitaxel sensitivity and hypoxia-inducible factor-1α expression in human ovarian cancer cells. Oncol Lett 6:795–800. 10.3892/ol.2013.1432 PMC3789026 24137413

[238] Yu X, Chen Y, Tian R, Li J, Li H, Lv T. miRNA-21 enhances chemoresistance to cisplatin in epithelial ovarian cancer by negatively regulating PTEN. Oncol Lett 14:1807–10. 10.3892/ol.2017.6324 PMC5529949 28789414

[239] Yang H, Kong W, He L, Zhao J-J, O’Donnell JD, Wang J. MicroRNA expression profiling in human ovarian cancer: miR-214 induces cell survival and cisplatin resistance by targeting PTEN. Cancer Res 68:425–33. 10.1158/0008-5472.CAN-07-2488 18199536

[240] Liu J, Chen W, Zhang H, Liu T, miR ZL. 214 targets the PTEN mediated PI3K/Akt signaling pathway and regulates cell proliferation and apoptosis in ovarian cancer. Oncol Lett. 10.3892/ol.2017.6953 PMC5661372 29113199

[241] Zhang Q, Zhang S. miR-214 promotes radioresistance in human ovarian cancer cells by targeting PETN. Biosci Rep. 10.1042/BSR20170327 PMC6434173 28559385

[242] Liu Y, Han S, Li Y, Liu Y, Zhang D, Li Y. MicroRNA-20a contributes to cisplatin-resistance and migration of OVCAR3 ovarian cancer cell line. Oncol Lett 14:1780–6. 10.3892/ol.2017.6348 PMC5529943 28789409

[243] Luo X, Dong Z, Chen Y, Yang L, Lai D. Enrichment of ovarian cancer stem-like cells is associated with epithelial to mesenchymal transition through an miRNA‐activated < scp > AKT pathway. Cell Prolif 46:436–46. 10.1111/cpr.12038 PMC6496712 23869765

[244] Amini-Farsani Z, Sangtarash MH, Shamsara M, Teimori H. MiR-221/222 promote chemoresistance to cisplatin in ovarian cancer cells by targeting PTEN/PI3K/AKT signaling pathway. Cytotechnology 70:203–13. 10.1007/s10616-017-0134-z PMC5809651 28887606

[245] Fu X, Tian J, Zhang L, Chen Y, Hao Q. Involvement of microRNA-93, a new regulator of pten/akt signaling pathway, in regulation of chemotherapeutic drug cisplatin chemosensitivity in ovarian cancer cells. FEBS Lett 586:1279–86. 10.1016/j.febslet.2012.03.006 22465665

[246] Zou YT, Gao JY, Wang HL, Wang Y, Wang H, Li PL. Downregulation of microRNA-630 inhibits cell proliferation and invasion and enhances chemosensitivity in human ovarian carcinoma. Genet Mol Res 14:8766–77. 10.4238/2015.July.31.25 26345808

[247] Shi X, Xiao L, Mao X, He J, Ding Y, Huang J. miR-205-5p Mediated Downregulation of PTEN Contributes to Cisplatin Resistance in C13K Human Ovarian Cancer Cells. Front Genet. Available from: https://www.frontiersin.org/article/10.3389/fgene.2018.00555/full 10.3389/fgene.2018.00555 PMC6253938 30510566

[248] Chen L, Zhang F, Sheng X-G, Zhang S-Q, Chen Y-T, Liu B-W. MicroRNA-106a regulates phosphatase and tensin homologue expression and promotes the proliferation and invasion of ovarian cancer cells. Oncol Rep 36:2135–41. 10.3892/or.2016.5010 27510094

[249] Huh JH, Kim TH, Kim K, Song J-A, Jung YJ, Jeong J-Y. Dysregulation of miR-106a and miR-591 confers Paclitaxel resistance to ovarian cancer. Br J Cancer. 109:452–61. 10.1038/bjc.2013.305 PMC3721386 23807165

[250] Zhu X, Shen H, Yin X, Yang M, Wei H, Chen Q. Macrophages derived exosomes deliver miR-223 to epithelial ovarian cancer cells to elicit a chemoresistant phenotype. J Exp Clin Cancer Res. 38. 10.1186/s13046-019-1095-1 PMC6377760 30770776

[251] MTM J, AWM JH, PF B, Ij KWF. E D. miR-141 regulates KEAP1 and modulates cisplatin sensitivity in ovarian cancer cells. Oncogene 32:4284–93. 10.1038/onc.2012.433 23045278

[252] Li T, Lin L, Liu Q, Gao W, Chen L, Sha C. Exosomal transfer of miR-429 confers chemoresistance in epithelial ovarian cancer. Am J Cancer Res 11:2124–41. PMC8167704 34094673

[253] Kamdar RD, Harrington BS, Attar E, Korrapati S, Shetty J, Zhao Y. NF-κB Signaling Modulates miR-452-5p and miR-335-5p Expression to Functionally Decrease Epithelial Ovarian Cancer Progression in Tumor-Initiating Cells. Int J Mol Sci. Available from: https://www.mdpi.com/1422-0067/24/9/7826 10.3390/ijms24097826 PMC10178396 37175530

[254] Jin P, Liu Y, Wang R. STAT3 regulated miR-216a promotes ovarian cancer proliferation and cisplatin resistance. Biosci Rep. 38. 10.1042/BSR20180547 PMC6131203 30061175

[255] Tan W-X, Sun G, Shangguan M-Y, Gui Z, Bao Y, Li Y-F. Novel role of LncRNA CHRF in cisplatin resistance of ovarian cancer is mediated by miR-10b induced EMT and STAT3 signaling. Sci Rep. 10. 10.1038/s41598-020-71153-0 PMC7478977 32901049

[256] Parikh A, Lee C, Joseph P, Marchini S, Baccarini A, Kolev V. microRNA-181a has a critical role in ovarian cancer progression through the regulation of the epithelial–mesenchymal transition. Nat Commun 5. 10.1038/ncomms3977 PMC3896774 24394555

[257] Li X, Gao X, Yuan J, Wang F, Xu X, Wang C. The miR-33a-5p/CROT axis mediates ovarian cancer cell behaviors and chemoresistance via the regulation of the TGF-β signal pathway. 10.3389/fendo.2022.950345 PMC9478117 36120434

[258] Mo Y, Leung LL, Mak CSL, Wang X, Chan W-S, Hui LMN. Tumor-secreted Exosomal miR-141 activates tumor-stroma interactions and controls premetastatic niche formation in ovarian cancer metastasis. Mol Cancer. 22. 10.1186/s12943-022-01703-9 PMC9827705 36624516

[259] Mohamed Z, Hassan MK, Okasha S, Mitamura T, Keshk S, Konno Y. miR-363 confers taxane resistance in ovarian cancer by targeting the Hippo pathway member, LATS2. Oncotarget 9:30053–65. 10.18632/oncotarget.25698 PMC6059020 30046387

[260] Xu M, Xiao J, Chen M, Yuan L, Li J, Shen H. MiR 149 5p promotes chemotherapeutic resistance in ovarian cancer via the inactivation of the Hippo signaling pathway. Int J Oncol. 10.3892/ijo.2018.4252 PMC5807033 29393390

[261] Arrighetti N, Cossa G, Cecco L, Stucchi S, Carenini N, Corna E. PKC-alpha modulation by miR-483-3p in platinum-resistant ovarian carcinoma cells. Toxicol Appl Pharmacol 310:9–19. 10.1016/j.taap.2016.08.005 27554045

[262] ZHAO H, BI T, QU Z, JIANG J, WANG CUIS. Y. Expression of miR-224-5p is associated with the original cisplatin resistance of ovarian papillary serous carcinoma. Oncol Rep 32:1003–12. 10.3892/or.2014.3311 25017423

[263] Zou D, Wang D, Li R, Tang Y, Yuan L, Long X. MiR-197 induces taxol resistance in human ovarian cancer cells by regulating NLK. Tumor Biol 36:6725–32. 10.1007/s13277-015-3365-7 25833695

[264] Gu Z-W, He Y-F, Wang W-J, Tian Q, Di W. MiR-1180 from bone marrow-derived mesenchymal stem cells induces Glycolysis and chemoresistance in ovarian cancer cells by upregulating the Wnt signaling pathway. J Zhejiang Univ B 20:219–37. 10.1631/jzus.B1800190 PMC6421125 30829010

